# Interaction between m6A and YAP1 mechanotransduction pathways is essential for mechanical memory and matrix remodeling in pancreatic cancer

**DOI:** 10.7150/ijbs.125330

**Published:** 2026-02-04

**Authors:** Jiaoshun Chen, Gengqiao Wang, Haoxiang Zhang, Qingyi Hu, Jun Zhao, Qiang Shen, Qingke Duan, Tao Yin

**Affiliations:** 1 Department of Pancreatic Surgery, Union Hospital, Tongji Medical College, Huazhong University of Science and Technology, Wuhan 430022, China.; 2 Sino-German Laboratory of Personalized Medicine for Pancreatic Cancer, Union Hospital, Tongji Medical College, Huazhong University of Science and Technology, Wuhan 430022, China.; 3 Shengli Clinical Medical College of Fujian Medical University, Fuzhou, 350001, China.; 4 Department of Hepatopancreatobiliary Surgery, Fujian Provincial Hospital, Fuzhou, 350001, China.; 5 Department of Breast and Thyroid Surgery, Union Hospital, Tongji Medical College, Wuhan 430022, China.; 6 Department of Anatomy, School of Basic Medicine, Tongji Medical College, Huazhong University of Science and Technology, Wuhan 430030, China.; 7 Department of Interdisciplinary Oncology, Louisiana State University Health Sciences Center, New Orleans, LA, United States.; 8 Department of Radiation Oncology, The Affiliated Cancer Hospital of Zhengzhou University & Henan Cancer Hospital, Zhengzhou 450008, China.

**Keywords:** pancreatic cancer, CD166-EGFR-LOXL2 axis, matrix stiffness, YAP1, METTL14, mechanical memory

## Abstract

Pancreatic cancer is a highly aggressive malignancy characterized by a progressively stiffened extracellular matrix, which promotes mechanical memory acquisition in cancer cells and facilitates malignant progression and metastasis. Despite its clinical significance, the mechanisms underlying matrix stiffening and mechanical memory formation remain poorly defined. This study demonstrates that a high-stiffness microenvironment induces mechanical memory in pancreatic tumor cells, which in further aggravates stromal remodeling and adversely affects prognosis. Under mechanically stiff conditions, pancreatic cancer cells exhibit pronounced enrichment of RNA modification-related and metabolic pathways, along with significantly increased m6A levels. Mechanistically, METTL14 enhances YAP1 expression through YTHDF3-mediated m6A-dependent translational regulation, while YAP1 in turn transcriptionally upregulates METTL14 via TEAD1, establishing a positive feedback loop that sustains mechanical memory. This METTL14-YAP1 axis activates CD166-EGFR-LOXL2 signaling, leading to enhanced collagen cross-linking and deposition, increased stromal stiffness, and maintenance of tumor stemness. These results identify the METTL14-YAP1 feedback loop as a core regulator of mechanical memory in pancreatic ductal adenocarcinoma, which drives stromal dysfunction and tumor progression through CD166-LOXL2 axis, and suggest targeting this loop as a potential therapeutic strategy to disrupt mechanical memory and ameliorate stiffness-induced remodeling.

## Introduction

Matrix stiffness is a critical parameter defining the physical characteristics of the tumor microenvironment. Progressively increasing stromal stiffness activates signaling cascades related to cell proliferation, migration, and invasion^1^. Changes in matrix stiffness further regulate immune cell recruitment and anti-tumor functions, modulating tumor growth^2^. Additionally, matrix stiffness significantly impacts intratumoral drug delivery efficiency and therapeutic responses^3^,^4^. Reports indicate that pancreatic ductal adenocarcinoma (PDAC) matrix stiffness is several-fold higher than adjacent healthy tissue, potentially contributing to its high invasiveness and poor prognosis^5^,^6^.

Emerging evidence indicates that sustained exposure to a high-stiffness matrix induces mechanical memory in tumor cells. This epigenetically-driven transcriptional reprogramming confers persistent pro-metastatic phenotypes—including enhanced migratory capacity, stemness, and chemoresistance—which are maintained even after dissemination to softer microenvironments, thereby accelerating metastatic progression and malignant evolution^7^,^8^. Critically, the establishment of tumor mechanical memory promotes further matrix stiffening, amplifying rigidity within the tumor microenvironment through a self-reinforcing feedback loop^9^.

Tumor stemness is widely recognized as a critical component in tumor progression. Stiffness and mechanical memory play essential roles in the establishment and maintenance of stemness, as ECM modulates the stem cell niche—its rigidity regulating key cellular processes including growth, survival, and homeostasis^8^,^10^. Tumor cells with stem-like properties possess self-renewal and multi-lineage differentiation capabilities, which drive tumor heterogeneity and serve as central factors in tumor recurrence, metastasis, and therapy resistance. Although biomechanical forces are known to exert a pivotal and dynamic influence on tumor biology, the underlying mechanisms through which extracellular stresses and mechanical memory regulate stemness to promote malignant progression remain poorly understood.

Yes-associated protein 1 (YAP1), a key transcriptional co-activator and central effector of the Hippo pathway, plays a fundamental role in regulating cell proliferation, differentiation, and apoptosis^11^,^12^. Notably, YAP1 serves as a critical mechanotransducer that converts microenvironmental biomechanical signals—particularly elevated matrix stiffness—into transcriptional programs which drive malignant phenotypes^12^,^13^. Furthermore, YAP1 acts as an intracellular mechanical rheostat, central to the formation and maintenance of mechanical memory in cancer cells. As mechanoresponsive transcriptional regulators, YAP1 transduce extracellular mechanical cues into sustained transcriptional outputs that promote a stem-like state^12^-^14^. Their activation is modulated by ECM stiffness and cellular contractility, enabling the encoding of prior mechanical exposures into persistent pro-oncogenic gene expression signatures. This mechanosensory function establishes YAP as essential mediators through which tumor cells retain a memory of past physical microenvironments, thereby enhancing stemness and supporting adaptive tumor progression.

N6-methyladenosine (m6A), one of the most abundant RNA modifications, serves as a pivotal regulator of RNA metabolism, influencing stability, translation, and splicing^15^. The dynamic regulation of m6A is orchestrated by writer (e.g., METTL3/14, WTAP), eraser (e.g., FTO, ALKBH5), and reader proteins^16^. In the context of tumor mechanobiology, m6A-mediated post-transcriptional modification of mRNAs encoding mechanosensitive factors—such as YAP/TAZ—modulates their stability and translational efficiency^17^,^18^. This mechanism facilitates the conversion of extracellular mechanical cues, like matrix stiffness, into sustained transcriptional outputs, thereby contributing to the establishment of mechanical memory in cancer cells. Such memory promotes acquisition of stem-like traits, enhances adaptive survival mechanisms, and confers therapy resistance, collectively driving malignant progression. Nevertheless, the crosstalk between m6A dysregulation and mechanotransduction pathways remains largely unexplored in pancreatic cancer.

The present study aims to elucidate the molecular mechanisms underlying stromal stiffening and the establishment of mechanical memory in PDAC, thereby providing new insights and a theoretical foundation for clinical diagnosis and treatment. We propose that increased matrix stiffness promotes the formation of an m6A-YAP1 positive feedback loop in tumor cells. This reprogrammed signaling pathway plays a critical role in sustaining mechanical memory and subsequently regulates key malignant traits, including stemness, metastatic dissemination, and disease progression. A deeper understanding of these mechanobiological interactions may unveil novel therapeutic opportunities.

## Materials and Methods

### Ethical approval

The Ethics Committee of Union Hospital, Tongji Medical College, Huazhong University of Science and Technology has approved this research. Patients signed the written informed consent before surgery.

The Animal Care Committee of Union Hospital, Tongji Medical College, Huazhong University of Science and Technology approved this study.

### Establishment of high-stiffness environment and mechanical memory cells

For the establishment of the high-stiffness environment cell line, we coated 10 cm culture dishes with polyacrylamide gels of different stiffness gradients (1 kPa, 10 kPa, 20 kPa). Cells were seeded in these dishes and maintained in DMEM supplemented with 10% fetal bovine serum, with medium changed daily. When the cell density reached approximately 80%, the cells were digested and passaged, with one-quarter of the cells reseeded into fresh dishes for continuous culture. This culturing and passaging protocol was maintained for 2 months to establish the high-stiffness environment-adapted pancreatic cancer cell line. For the establishment of tumor mechanical memory, we referred to the method described by Killaars et al.^8^,^19^. PANC1 and BxPC-3 cells were initially cultured on a stiff substrate (20 kPa) to establish a stable mechanical memory. For the mechanical memory group, cells were cultured on the stiff substrate for 10 days, inducing a memory state that persists for approximately 5 days. In contrast, the non-memory group was cultured on the same stiff substrate for only 3 days. Following this priming phase, cells from both groups were transferred to a soft substrate (1 kPa) for continued culture.

### m6A dot-blot

Total RNA was extracted from cells, and 1 μg aliquots were dotted onto nitrocellulose membranes in replicates. RNA was cross-linked with 254 nm UV light for 5 min. Skim milk (5%) was used to block membranes at 20℃ for 1 h, followed by incubation with a primary antibody against m6A (1:1000 dilution) overnight at 4 °C. Membranes were washed three times with TBST for 10 min each and incubated with a horseradish peroxidase-conjugated anti-rabbit secondary antibody (1:5000 dilution) at room temperature for 1 h, followed by three TBST washes. ECL solution was added for 5 min before exposure and imaging.

### MeRIP-qPCR

MeRIP-qPCR was performed according to the EpiQuik CUT & RUN m6A RNA Enrichment (MeRIP) kit (Epigentek, P-9018) protocol. Briefly, an immunocapture solution was prepared by rotating the reagents in 0.2 mL PCR tubes at room temperature for 90 min. Nucleic acid digestion enhancer (NDE; 10 μL) and cleavage enzyme mix (CEM; 2 μL) were added to each tube and incubated at room temperature for 4 min. The tubes were placed on a magnetic device (Beyotime, China) until the solution was clear (approximately 2 min). The supernatant was collected and discarded. Samples were washed thrice with 150 μL wash buffer and once with 150 μL protein digestion buffer. Subsequently, 20 μL Proteinase K digestion solution was added, mixed, and incubated at 55 °C for 15 min in a thermal cycler without a heated lid. RNA purification solution and 100% ethanol were subsequently added to the samples, which were then resuspended and washed with RNA-binding beads and vortexed. Treated beads were processed with 13 μL elution buffer at room temperature for 5 min to release RNA from beads. Finally, 13 μL from each sample was transferred into new 0.2 mL PCR tubes for immediate use or storage at -20 °C.

### Chromatin immunoprecipitation (ChIP) assay

Approximately 1 × 10^7^ cells were harvested and resuspended in lysis buffer containing protease and phosphatase inhibitors, followed by a 10-min incubation on ice. Formaldehyde was then added to a final concentration of 1% for a 10-min incubation at room temperature to crosslink proteins to DNA, quenched with glycine. Cells were pelleted, resuspended in nuclear lysis buffer, and incubated on ice for 10 min. The lysates were then sonicated to shear DNA between 200-1000 base pairs; shearing efficiency was verified via agarose gel electrophoresis. The sheared chromatin was precleared with protein A or G beads, then incubated overnight at 4 °C with gentle rotation using specific antibodies against the target protein. Protein A or G beads were added to the chromatin-antibody mixture and incubated for 2 h at 4 °C. Following multiple washes with various wash buffers to remove non-specifically bound materials, DNA-protein complexes were eluted from the beads using an elution buffer. NaCl was added to the eluted complexes and incubated overnight at 65 °C to reverse DNA-protein crosslinks. DNA was subsequently extracted and purified using phenol-chloroform extraction and ethanol precipitation. The precipitated DNA was analyzed using quantitative PCR, microarray, or next-generation sequencing to identify DNA sequences associated with the protein of interest.

### SELECT-m6A

To detect and identify m6A modification sites, we used the Epi-SELECT™ m6A Site Identification Kit (Guangzhou Epibiotek Co., Ltd., Guangzhou, China, R202106M-02). FTO treatment was conducted at 37 °C for 30 min, using 5 μL FTO enzyme and 10 μL 5× FTO reaction buffer to remove m6A modifications from the RNA template. Subsequently, first-strand cDNA synthesis was carried out, including RNA template denaturation and a reverse transcription reaction, employing specific volumes of RT buffer, DTT, RT primer, and dNTPs. Next, gene-specific quantification was performed via qPCR to ensure consistent initial RNA levels between the experimental and control groups. The qPCR reaction system comprised 1 μL cDNA template, 5 μL 2× ChamQ Universal SYBR qPCR Master Mix, and 0.2 μL gene-specific primers. The qPCR reaction conditions included a pre-denaturation step at 95 °C for 30 s, followed by 40 cycles, each consisting of 5 s at 95 °C and 30 s at 60 °C. Finally, melting curve analyses were conducted to confirm the specificity of the amplification.

### RNA pull-down assay

Biotinylated RNA probes were transcribed *in vitro* (Bersin Bio, China). Cell lysates were prepared and mixed with biotinylated RNA by gentle rotation for 1 h to allow binding. Washed streptavidin beads were added and incubated for 3 h at 4 °C to capture biotinylated RNA. After three washes, beads were centrifuged at 8,000 rpm and 4 °C, whereafter the supernatant was removed. Proteins were denatured and subjected to sodium dodecyl sulfate-polyacrylamide gel electrophoresis (SDS-PAGE), followed by western blotting to detect RNA-bound proteins.

### RNA half-life

Actinomycin D was added at a 5 μg/mL final concentration to exponentially growing cells. At various time points (0, 2, 4, 6, and 8 h) after actinomycin D treatment, cells were collected, and total RNA was extracted using a kit (Novogene, China). RNA concentration and purity were determined using a microspectrophotometer. Genomic DNA contamination was removed by treating with DNase I. cDNA was synthesized via reverse transcription, and target RNA levels were measured at each time point using real-time quantitative PCR (qPCR). RNA degradation kinetics were plotted to calculate RNA half-life using linear regression analysis.

### Dual-luciferase reporter assay

Reporter plasmids containing the luciferase gene downstream of the target gene promoter were constructed (Jikai, China). Reporter and internal control plasmids were co-transfected into the cells and cultured for 24 h before being transformed into complete medium. At 48 h post-transfection, the culture medium was removed, and the cells were washed with PBS. Cell lysis buffer was then added. The supernatant was collected after centrifugation, and *Renilla* luciferase substrates were added. Fluorescence and luminescence values were measured using a microplate reader to calculate the relative luciferase units of the reporter. Negative and positive controls were included with three to five technical replicates per sample. The promoter and transcriptional activities of the target genes were indirectly assessed.

### Sphere formation assay

Cells were cultured in serum-free DMEM/F12 medium supplemented with 2% B-27 (#17504044, Gibco, USA), heparin (4 μg/mL), epidermal growth factor (20 ng/mL; #236-EG-200, R&D, USA), and fibroblast growth factor (20 ng/mL; #AF-100-18C, PEPROTECH, USA). DMEM/F12 medium with varying glucose concentrations was prepared by mixing equal volumes of low-glucose DMEM (Gibco, USA) and F12 medium (Gibco, USA) and adding D-glucose (#G116304, Aladdin Industrial Corporation, Shanghai, China). The assay was performed in 10 ultra-low attachment 6-well plates at 500 cells/well. After 7 days, sphere formation was analyzed using phase-contrast microscopy.

### Mouse orthotopic model with graded stiffness

All animal studies were approved by the Animal Care and Use Committee of Tongji Medical College, Huazhong University of Science and Technology, and were conducted in accordance with the National Research Council's Guide for the Care and Use of Laboratory Animals at specific pathogen-free facilities. The modeling method is described in detail in our previous publication^36^. Briefly, KPC cells were thoroughly mixed in a GelMA solution (5 × 10^5^ cells/20 μL) and injected into the exposed pancreas through a left flank incision. The GelMA-KPC cell suspension was photo-cross-linked using a 405 nm light curing system (EFL-LS-1601-405, Suzhou Yongqinquan Intelligent Equipment Co., Ltd., China) within 30 s. The needle was withdrawn after cross-linking, and the abdominal wall was sutured layer-by-layer with 7-0 silk. All procedures followed institutional animal ethics guidelines.

### Mouse subcutaneous tumor experimental method

Six-week-old male C57 mice (n=5/group) were used. After KPC cells were cultured on substrates with a stiffness of 20 kPa or 1 kPa for a certain period of time, they were used for subcutaneous tumor experiments. Log-phase KPC cells (1×10⁶ cells/100μL) suspended in PBS were subcutaneously injected into the right axilla of each mouse. Tumor volume (V=0.5×length×width²) was measured every 3 days with a vernier caliper. After 21 days, mice were euthanized. Tumors were excised, weighed, and stored at -80°C for subsequent analysis. All procedures followed institutional animal ethics guidelines.

### Mouse liver metastasis method

Six-week-old C57 mice (n=6/group) were anesthetized. Log-phase KPC cells (1×10⁵ cells/50μL PBS) were injected into the spleen. After hemostasis, incisions were sutured. Mice were euthanized on day 28. Livers were excised to count metastatic nodules and weighed. All procedures followed animal ethics guidelines.

### Tumor drug perfusion experiment

Four hours before euthanizing the mice, 200 μL of 40 mg/mL FITC-dextran with a molecular weight of 10 kDa was injected via the tail vein. After euthanasia, the tumors were excised, and frozen sections were prepared. The sections were stained with 4,6-diamidino-2-phenylindole (DAPI) and images were collected. ImageJ software (Fiji) was used to quantify the integrated density signals of the images.

### *In vivo* imaging of mice

Luciferase signals were imaged to monitor tumor progression. The luciferase substrate was injected intraperitoneally into the mice, and 15 min later, the mice were anesthetized with isoflurane. The signal was acquired with open filters and small binning. Radiance was used as a measure of signal intensity to confirm tumor presence.

### Immunofluorescence

The cells were digested, washed with PBS, seeded on coverslips, fixed with 4% paraformaldehyde at room temperature for 15 min, and permeabilized with 0.1% Triton X-100 for 5 min. After blocking at room temperature for 30 min, the cells were incubated overnight at 4 °C with the primary antibody at optimized dilution. The next day, the cells were washed three times with PBS for 5 min each and protected from light. Fluorescent-labeled secondary antibodies were incubated for 1 h at room temperature. The cells were stained with DAPI before three final PBS washes. Slides were mounted with an antifade reagent and covered using coverslips. Fluorescent images were acquired using a confocal microscope with the appropriate filters.

### Matrix cross-linking experiment

Ten million pancreatic cancer cells from culture dishes were digested and resuspended in 500 μL type I collagen fibers (Thermo Fisher, USA). The mixture was then thoroughly mixed by pipetting. Subsequently, the mixture was naturally spread into a 6-well plate and incubated at 37 °C for 30 min until the collagen gel formed. After gelation, 3 mL cell culture medium was added to each well. Photographs were taken daily to record the size and change medium. After 5 days, samples were subjected to electron microscopy to observe pore size and cross-linking.

### Matrix stiffness measurement

Twenty million pancreatic cancer cells were digested from culture dishes and resuspended in 1000 μL type I collagen fibers (Thermo Fisher, USA). The mixture was then thoroughly mixed by pipetting. The above system was spread into 24-well plates and incubated at 37 °C for 30 min. After collagen gelation, gels were transferred to 10 cm culture dishes. After culturing for 5 days, matrix stiffness was measured using a universal mechanical tester, and a stiffness curve was generated.

### Data collection and ethics

Data from 100 patients with PDAC who underwent endoscopic ultrasound examinations were collected from the Union Hospital, Huazhong University of Science and Technology, Wuhan, China. This study was approved by the Ethics Committee of Tongji Medical College, Huazhong University of Science and Technology, Wuhan, China.

### Statistical analysis

Statistical analyses were conducted using SPSS software (version 21.0; IBM, NY, USA). Data are presented as the mean ± standard deviation (SD). Two-tailed Student's *t-*test or Wilcoxon rank-sum test was used for comparisons between two groups. One-way ANOVA was used for comparisons among the three groups. All experiments were performed in triplicate. Statistical significance was set at *P* < 0.05.

### Resource availability

The raw data supporting the conclusions of this article will be made available by the authors without undue reservation.

## Results

### PDAC stiffness is associated with clinical progression and prognosis

**Figure 1:**
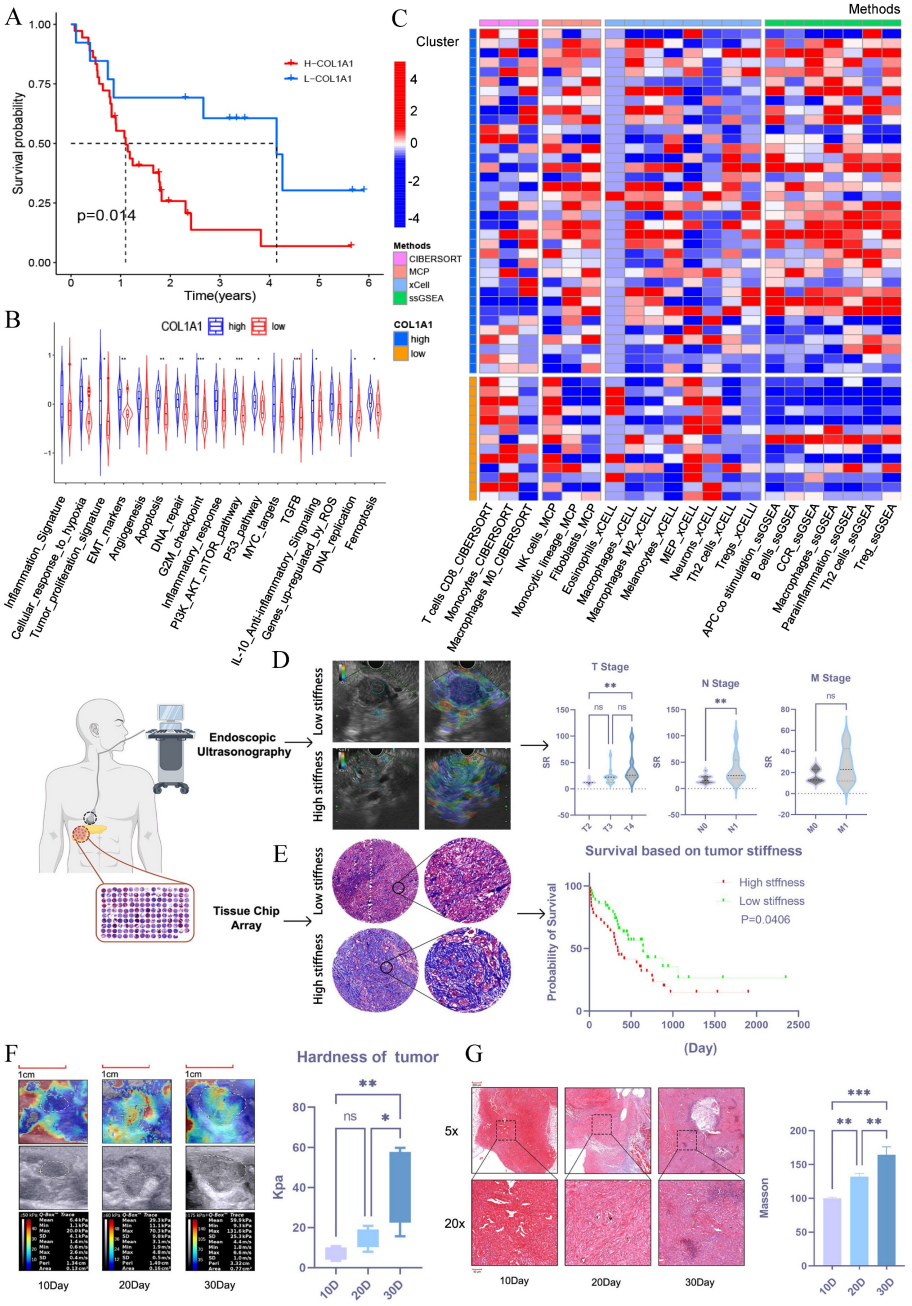
**PDAC stiffness is associated with clinical progression and prognosis.** (A) Survival analysis between the high-stiffness group and the low-stiffness group in the GSE78229 dataset. (B) KEGG pathway enrichment analysis of DEGs between the high-stiffness and low-stiffness groups in the GSE78229 dataset. (C) Analysis of immune infiltration between the high-stiffness and low-stiffness groups in the GSE78229 dataset. (D) Correlations between tumor stiffness and T stage, N stage, and M stage, respectively; n = 50. (E) Prognosis of patients with high and low stiffness, as determined using tissue microarrays; n = 80. (F) Changes in stiffness during the progression of *in situ* pancreatic tumors in mice, as detected via elastography; n = 15. (G) Results of Masson's staining of *in situ* pancreatic tumors in mice; n = 15. Data are presented as mean ± standard of error (SD). Statistical significance was determined using ANOVA with post-hoc Tukey multiple comparison, * P < 0.05, ** P < 0.01, *** *P* < 0.0001, n.s.: not significant.

To explore whether tumor stiffness is associated with prognosis in pancreatic cancer, we conducted bioinformatic analysis using the GSE78229 and GSE62452 datasets from GEO. Tumor stiffness was inferred based on the expression level of COL1A1^20^, and pancreatic cancer patients were divided into high-stiffness and low-stiffness groups. Prognostic analysis revealed that patients with high-stiffness pancreatic cancer had worse outcomes (Figure 1A). KEGG pathway analysis of differentially expressed genes between the high-stiffness and low-stiffness groups indicated significant enrichment in tumor proliferation, EMT, and PI3K-AKT pathways in the high-stiffness group, suggesting that high stiffness may be associated with poor prognosis (Figure 1B). Through immune infiltration analysis, we also found that CD8+ T cells were significantly downregulated in high-stiffness pancreatic cancer, while immunosuppressive cells such as fibroblasts, M2 macrophages, and Tregs were significantly upregulated, indicating that high stiffness might promote the formation of an immunosuppressive microenvironment (Figure 1C). These results suggest that the matrix stiffness of pancreatic cancer may be a key factor affecting prognosis. To validate this notion, we collected 50 PDAC samples from endoscopic ultrasonography procedures conducted at the Union Hospital of Tongji Medical College, Huazhong University of Science and Technology between 2022 and 2023. We used the strain ratio (SR) to assess the stiffness of pancreatic cancer. These samples underwent ultrasound elastography to analyze the correlation between stromal stiffness and tumor TNM staging. The matrix stiffness was significantly high in large-sized tumors (T stage) or in those with more affected lymph nodes (N stage) but remained similar across various metastatic burdens (M stage) (Figure 1D). Further validation using a tissue microarray of 80 PDAC tumors also confirmed that tumors with high stroma stiffness, as assessed by Masson's trichrome staining, correlated with short patient survival (Figure 1E). Therefore, it was established that a stiff stroma facilitated PDAC progression in patients.

### PDAC stiffness gradually increases during tumor progression

We then investigated how stroma stiffness changes during tumor progression. A murine orthotopic KPC tumor model was established, and its stiffness was measured via ultrasound elastography on the 10th, 20th, and 30th day after inoculation. The Young's modulus of stroma steadily increased from 3.4-10.7 kPa on day 10 to 7.9-20.8 kPa on day 20, reaching as high as 15.7-59.5 kPa on day 30 (Figure 1F). The increased stroma stiffness was accompanied by a gradual increase in tumor fibrosis, as shown by Masson trichrome staining of tissue sections from the corresponding time points (Figure 1G). Overall, these results show that there was a significant increase in stroma stiffness with PDAC progression.

### Mechanical memory accelerates tumor malignant progression and metastasis

**Figure 2:**
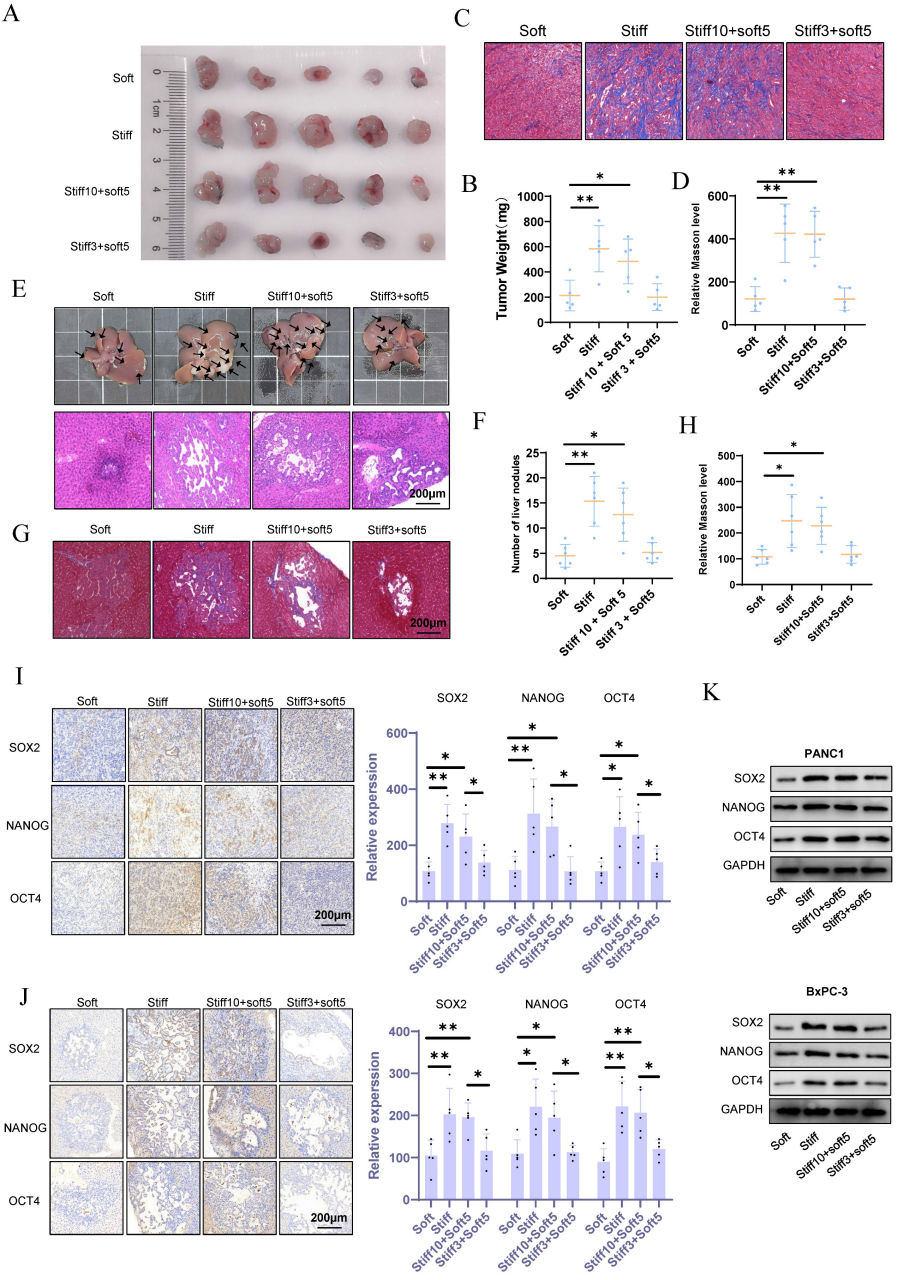
** Mechanical memory accelerates tumor malignant progression and metastasis.** (A-B) KPC cells were cultured at 20 kPa for 10 days (to induce mechanical memory formation) or 3 days (without mechanical memory formation), followed by transfer to a 1 kPa culture condition for 5 days. Subsequently, subcutaneous tumor implantation was performed, and the volume (A) and weight (B) of subcutaneous tumors in each group were measured; n = 5. (C-D) Masson's trichrome staining results (C) of mouse subcutaneous tumors and the statistical analysis of the proportion of positive area (D); n = 5. (E-F) After KPC cells were cultured in a 1 kPa or a 20 kPa for a certain period to acquire mechanical memory, a liver metastasis model was established (E), and the number of liver metastatic tumors in each group was counted (F); n = 6. (G-H) Masson's trichrome staining results (G) of mouse liver metastatic tumors and the statistical analysis of the proportion of positive area (H); n = 6. (I-J) The maintenance effect of mechanical memory on cancer stemness in subcutaneous tumors (I) and liver metastatic tumors (J) was evaluated by analyzing the expression of NANOG, SOX2 and OCT4 through IHC staining; n = 5. The PANC-1 and BxPC-3 were cultured at 20 kPa for 10 days (to induce mechanical memory formation) or 3 days (without mechanical memory formation), followed by transfer to a 1 kPa culture condition for 5 days, and the expression level of CD166, METTL14 and YAP1 were detected by western blotting; n = 3. Data are presented as mean ± standard of error (SD). Statistical significance was determined using ANOVA with post-hoc Tukey multiple comparison, * P < 0.05, ** P < 0.01, *** *P* < 0.0001, n.s.: not significant.

The pathophysiological stiffening of the tumor microenvironment prompts the acquisition of cell mechanical memory. To investigate the functional consequences of such memory on malignant progression and metastatic spread, we applied the mechano-priming protocol developed by Killaars *et al.*^8^,^19^. Immunofluorescence analysis of F-actin revealed that KPC preconditioned on stiff (20 kPa) substrates for 10 days sustained an ordered cytoskeletal morphology after being transferred to soft (1 kPa) substrates (stiff10-soft5). In contrast, cells cultured on stiff substrates for only 3 days (stiff3-soft5) showed complete cytoskeletal reversion (Fig. S1A), confirming the successful induction of mechanical memory. In murine subcutaneous tumor models, mechanical memory markedly accelerated malignant progression and exacerbated stromal fibrosis (Fig. 2A-D). Orthotopic liver metastasis models further demonstrated that mechanical memory enhanced the efficiency of metastatic colonization and promoted fibrotic remodeling within metastatic niches (Fig. 2E-H). Collectively, these results identify tumor mechanical memory as a pivotal biomechanical driver of aggressive disease progression, mediated through the coordinated reprogramming of stiffness microenvironments.

### Mechanical memory sustains oncogenic stemness in tumor cells

Tumor stemness constitutes a pivotal driver of malignant progression by mediating critical pathological processes including tumor initiation, metastasis, therapeutic resistance, and recurrence. However, the regulatory mechanism through which mechanical memory governs tumor stemness remains incompletely characterized. Our results demonstrate that mechanical memory persistently maintains elevated expression of pluripotency transcription factors (Nanog, Oct4, Sox2) (Fig. 2I). This effect extends to metastatic niches, as evidenced by sustained stemness signatures detected within hepatic metastases (Fig. 2J). In vitro studies further establish that pancreatic cancer cell lines PANC1 and BxPC-3 cultured on stiff substrates (20 kPa) exhibit significantly upregulated pluripotency factor expression (Nanog/Oct4/Sox2) compared to those on soft matrices (1 kPa). Notably, tumor cells retaining mechanical memory continue to preserve stemness characteristics following mechanical stimulus withdrawal (Fig. 2K and S1B).

### METTL14-mediated RNA m6A modification plays a significant role in the regulation of PDAC rigidity and impacts prognosis

**Figure 3:**
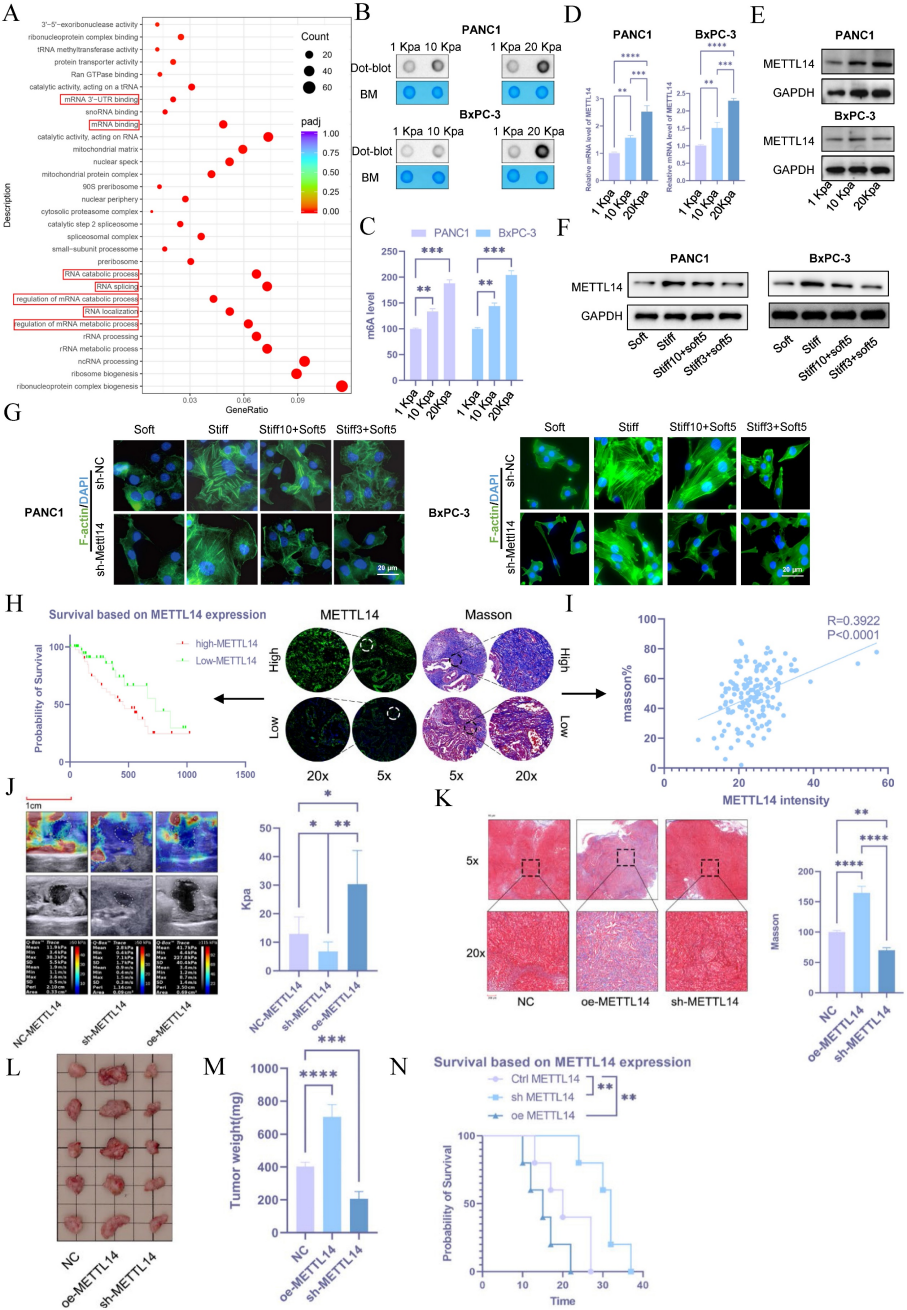
** METTL14-mediated RNA m6A modification plays a significant role in the regulation of PDAC rigidity and impacts prognosis.** (A) KEGG pathway analysis of differentially expressed genes in BxPC-3 cells cultured in 20 kPa and 1 kPa stiffness environments. (B) ME-dot blot assay to assess m6A methylation levels in pancreatic cancer cells under different stiffness conditions; n = 3. (C) ME-ELISA assay to assess m6A methylation levels in pancreatic cancer cells under different stiffness conditions; n = 3. (D-E) PCR (D) and western blot (E) analyses for METTL14 expression levels under varying matrix stiffness conditions; n = 3. (F) To evaluate the role of tumor mechanical memory in maintaining METTL14 expression, PANC-1 and BxPC-3 cells were first cultured at 20 kPa for 10 days (to induce mechanical memory formation) or 3 days (without mechanical memory formation). Subsequently, the cells were transferred to a 1 kPa culture environment for 5 days, and the expression level of METTL14 was detected by western blotting; n = 3. (G) To investigate the promoting effect of METTL14 on the formation and maintenance of tumor mechanical memory, METTL14 was knocked out in PANC-1 and BxPC-3 cells first. Then cells were then cultured at 20 kPa for 10 days (to induce mechanical memory formation) or 3 days (without mechanical memory formation), followed by transfer to a 1 kPa culture condition for 5 days. To assess the role of METTL14 in tumor cytoskeletal remodeling mediated by tumor mechanical memory, F-actin immunofluorescence staining was performed to observe tumor cytoskeletal rearrangement; n = 3. (H) Tissue microarray-based immunofluorescence analysis of the relationship between METTL14 and pancreatic cancer prognosis. (I) Correlation between METTL14 and matrix stiffness as analyzed via Masson staining and immunofluorescence based on tissue microarrays. (J-K) Elastography (J) and Masson staining (K) assessing the regulatory role of METTL14 in matrix stiffness in a mouse model; n = 5. (L-M) Tumor size (L) and weight (M) *in situ* for mouse xenografts with NC, METTL14 overexpression, and METTL14 knockdown; n = 5. (N) Survival analysis of mice with NC, METTL14 overexpression, and METTL14 knockdown; n = 5. Data are presented as mean ± standard of error (SD). Statistical significance was determined using ANOVA with post-hoc Tukey multiple comparison, * P < 0.05, ** P < 0.01, *** *P* < 0.0001, n.s.: not significant.

To investigate the mechanisms of stromal stiffness formation in pancreatic cancer, transcriptome sequencing was performed on cells cultured on 10 kPa and 20 kPa substrates compared with control BxPC-3 cells (1 kPa). Differentially expressed genes between the 20 kPa and 1 kPa groups were predominantly enriched in RNA modification-related pathways, including regulation of mRNA metabolic processes, mRNA degradation, mRNA binding, mRNA 3'-UTR binding, and control of RNA stability (Figure 3A), suggesting a potential link between stromal stiffness and RNA modifications. Given that m6A is the most prevalent RNA modification in eukaryotes, we hypothesized its involvement in this process.

Using m6A dot blot assays, we detected elevated m6A levels in PANC1 and BxPC-3 cells cultured on high-stiffness substrates compared to those on low-stiffness substrates (Figures 3B and 3C). As m6A levels are regulated by specific writers and erasers, we evaluated the expression of m6A-related molecules under 1, 10, and 20 kPa conditions via PCR and western blot. METTL14 exhibited the most pronounced upregulation under high-stiffness conditions (Figures 3D, 3E, and S1C, S1D). Notably, tumor cells with established mechanical memory sustained high METTL14 expression even after transfer to a soft environment (Figure 3F and S1E). F-act staining revealed that such cells maintained an organized cytoskeleton in soft conditions, whereas METTL14 inhibition disrupted mechanical memory (Figure 3G and S1F). In subcutaneous and liver metastasis models, METTL14 expression was elevated in cells cultured on stiff matrices and in those retaining mechanical memory (Figures S1G and S1H).

These results underscore the critical role of METTL14 in the formation and maintenance of tumor mechanical memory. Using tissue microarrays, immunofluorescence staining of METTL14 correlated with poor patient prognosis (Figure 3H). Furthermore, METTL14 expression positively correlated with stromal stiffness per Masson staining (Figure 3I), suggesting that METTL14 may promote stromal stiffness and influence prognosis in PDAC by sustaining mechanical memory.

To test this, we generated METTL14-knockdown (sh-METTL14) and overexpressing (oe-METTL14) KPC cell lines via lentiviral transduction, with efficiency verified by western blot (Figure S2A). In orthotopic models in C57 mice, elastography on day 20 showed that METTL14 knockdown reduced tumor stiffness, while overexpression increased it (Figure 3J). Masson's staining confirmed decreased fibrosis with METTL14 knockdown and increased fibrosis with overexpression (Figure 3K). METTL14 knockdown suppressed tumor growth, whereas overexpression promoted it (Figures 3L and 3M). Accordingly, METTL14 overexpression shortened overall survival, while knockdown extended it (Figure 3N).

### METTL14 regulates stromal stiffness in PDAC through YAP1

**Figure 4:**
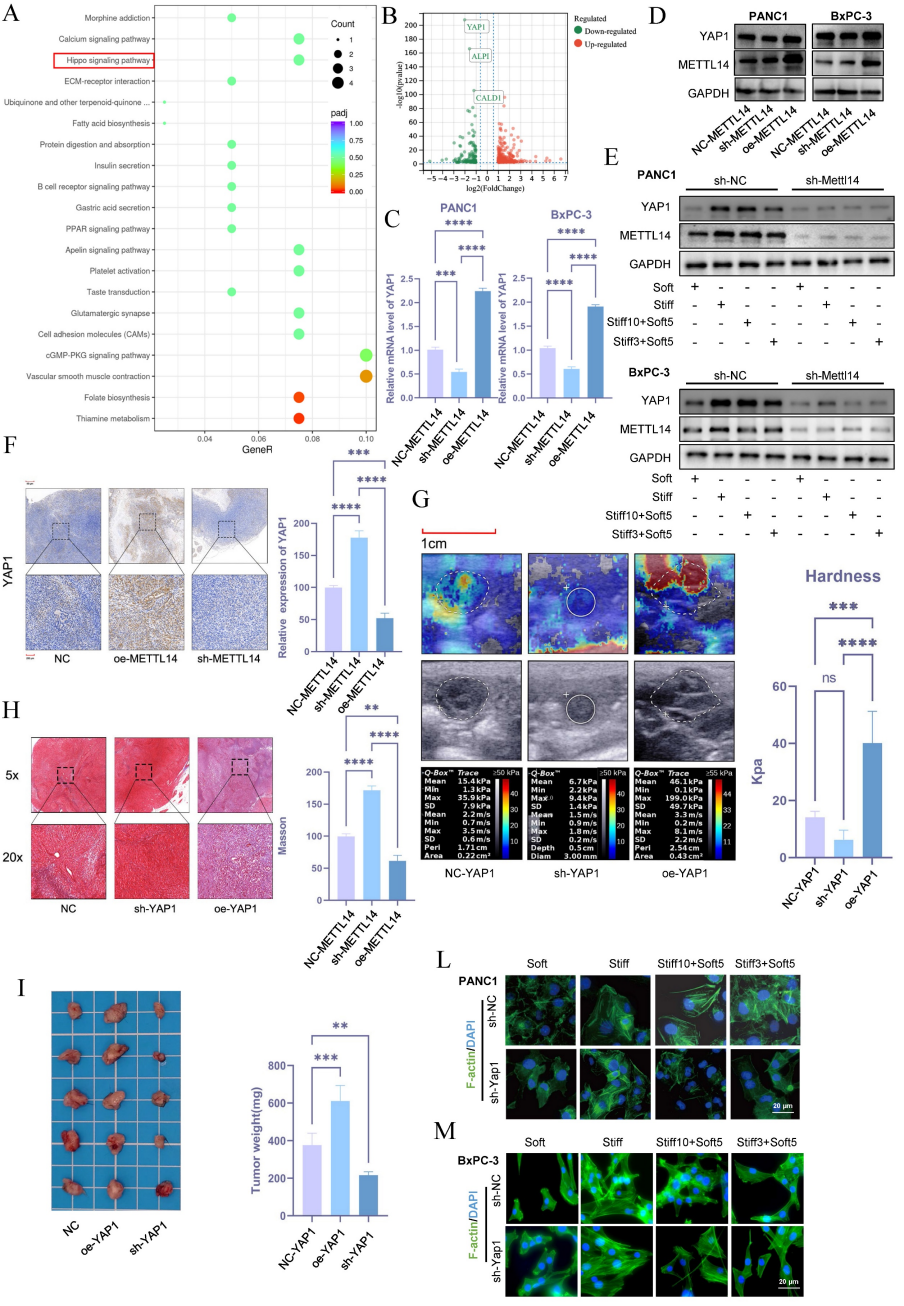
**METTL14 Regulates Stromal Stiffness in PDAC through YAP1.** (A) KEGG pathway analysis of differentially expressed genes in NC\METTL14 knockdown BxPC-3 cells. (B)Volcano plot of differentially expressed genes upon METTL14 knockdown. (C-D) PCR (C) and Western blot (D) analyses of YAP1 expression levels in pancreatic cancer cell lines with NC, METTL14 knockdown, and METTL14 overexpression; n = 3. (E) METTL14 was knocked out in PANC-1 and BxPC-3 cells first. Then cells were then cultured at 20 kPa for 10 days (to induce mechanical memory formation) or 3 days (without mechanical memory formation), followed by transfer to a 1 kPa culture condition for 5 days, and the expression level of METTL14 and YAP1 were detected by western blotting; n = 3. (F) Immunohistochemical analysis of YAP1 expression in mouse xenografts with NC, METTL14 knockdown, and METTL14 overexpression. (G-H) Elastography (G) and Masson staining (H) assessing the regulatory role of YAP1 on matrix stiffness in a mouse model. (I) Tumor size and weight *in situ* in mouse xenografts with NC, YAP1 overexpression, and YAP1 knockdown; n = 5. (L-M) To investigate the promoting effect of YAP1on the formation and maintenance of tumor mechanical memory, YAP1 was knocked out in PANC-1 and BxPC-3 cells first. Then cells were then cultured at 20 kPa for 10 days (to induce mechanical memory formation) or 3 days (without mechanical memory formation), followed by transfer to a 1 kPa culture condition for 5 days. To assess the role of YAP1 in tumor cytoskeletal remodeling mediated by tumor mechanical memory, F-actin immunofluorescence staining was performed to observe tumor cytoskeletal rearrangement; n = 3. Data are presented as mean ± standard of error (SD). Statistical significance was determined using ANOVA with post-hoc Tukey multiple comparison, * P < 0.05, ** P < 0.01, *** *P* < 0.0001, n.s.: not significant.

To investigate the role of METTL14 in stromal stiffness and prognosis of PDAC, we performed RNA sequencing in control and METTL14-knockdown BxPC-3 cells. Subsequent KEGG pathway analysis revealed that METTL14 suppression significantly down-regulated genes involved in the Hippo signaling pathway (Figure 4A). The Hippo pathway is known to regulate organ size and plays a central role in cancer development. Notably, the nuclear translocation of its effectors YAP/TAZ is influenced by mechanical cues such as stromal stiffness and mechanical memory. Among the downregulated genes, YAP1 showed the most significant reduction upon METTL14 knockdown (Figure 4B), suggesting that METTL14 may influence stromal stiffness and mechanical memory in PDAC via YAP1.

Consistent with this, METTL14 knockdown decreased both mRNA and protein levels of YAP1, while its overexpression elevated YAP1 expression (Figure 4C, D and S2B). Mechanical memory sustained YAP1 expression while this effect counteracted by METTL14 inhibition (Figure 4E and S2C). Immunohistochemistry further confirmed that METTL14 knockdown reduced YAP1 expression, whereas overexpression increased it (Figure 4F).

To assess whether YAP1 contributes to stromal stiffening in PDAC, we established YAP1-knockdown and overexpressing KPC cell lines and used them to generate orthotopic tumors in C57 mice. Elastography on day 20 showed that YAP1 knockdown reduced tumor stiffness, while YAP1 overexpression increased it (Figure 4G). Masson's staining corroborated YAP1's role in regulating stromal stiffness (Figure 4H). YAP1 knockdown also suppressed tumor growth, while overexpression promoted it (Figure 4I). Moreover, YAP1 overexpression shortened overall survival in mice, whereas knockdown extended it (Figure S2D). In vitro experiments indicated that stiffness and mechanical memory maintained YAP1 expression, and YAP1 knockdown abolished stiffness- and memory-driven YAP1 induction (Figure 4L-M, S2E). These findings underscore the critical role of YAP1 in mediating mechanical memory and stromal stiffness in PDAC.

### METTL14 upregulates YAP1 through a m6A-YTHDF3-dependent pathway

**Figure 5:**
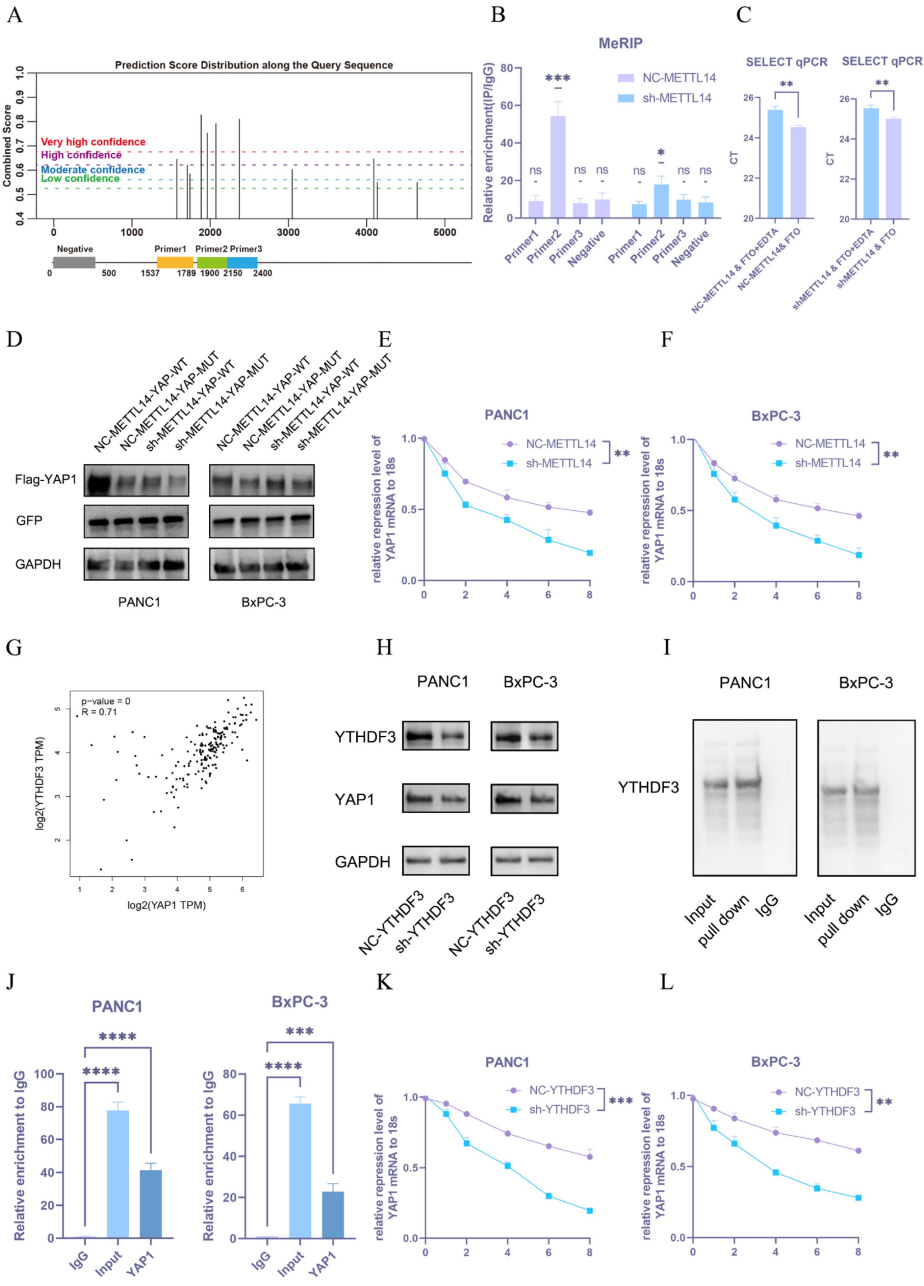
**METTL14 upregulates YAP1 through a m6A-YTHDF3-dependent pathway.** (A) Predicted m6A modification sites on YAP1 mRNA by the SRAMP database and a schematic of primer design. (B) Results of the MeRIP-qPCR experiment on predicted m6A fragments in YAP1 mRNA; n = 3. (C) Results of single-base m6A modification detection in NC-METTL14 and sh-METTL14 cells; n = 3. (D) Western blot detection results of YAP1 expression after m6A site mutation; n = 3. (E, F) Results of PCR detection of YAP1 mRNA stability after METTL14 knockdown in PANC1 (E) and BxPC-3 (F). Analysis of the correlation between YAP1 and YTHDF3 expression in pancreatic cancer using the TCGA database. Western blot detection results of YAP1 expression after YTHDF3 knockdown in PANC1 and BxPC-3 cells; n = 3. Results of METTL14 RNA pull-down experiment in PANC1 and BxPC-3 cell lines; n = 3. RIP-qPCR analysis of the interaction between YTHDF3 and YAP1 mRNA; n = 3. (K, L) Results of PCR detection of YAP1 mRNA stability after YTHDF3 knockdown in PANC1 (K) and BxPC-3 (L) cells. Data are presented as mean ± standard of error (SD). Statistical significance was determined using ANOVA with post-hoc Tukey multiple comparison, * P < 0.05, ** P < 0.01, *** *P* < 0.0001, n.s.: not significant.

To determine whether METTL14 regulates YAP1 expression in an m6A-dependent manner, we utilized the SRAMP database to predict potential m6A modification sites on YAP1 mRNA and designed primers for MeRIP-qPCR (Figure 5A). Four primer pairs were designed for the MeRIP assay. The results revealed significant amplification with primer 2, indicating m6A modification within that sequence (Figure 5B). Three potential m6A sites were identified in the region amplified by primer 2 and further verified using the m6A Select Single-Base Kit. The site with the highest prediction score was confirmed to be methylated (Figure 5C).

We subsequently constructed an overexpression plasmid carrying a mutated version of the YAP1 m6A site (YAP1-m6Aᴹᵁᵀ) with an upstream flag tag. Both this mutant and the wild-type YAP1 (YAP1-WT) plasmid were transfected into NC-METTL14 and sh-METTL14 cells, with GFP used as a transfection control to ensure consistent efficiency. YAP1 expression was significantly downregulated following site-directed mutation (Figure 5D and S2F), supporting the hypothesis that METTL14 regulates YAP1 expression via m6A methylation at this specific site.

Given that YAP1 mRNA levels remained largely unchanged upon METTL14 knockdown and considering the enrichment of stiffness-related genes in RNA metabolic pathways (Figure 2A), we hypothesized that METTL14 might modulate YAP1 mRNA stability. To test this, NC-METTL14 and sh-METTL14 cells were treated with actinomycin D, and YAP1 mRNA expression was monitored over 0-8 hours. METTL14 knockdown markedly reduced the half-life of YAP1 mRNA (Figure 5E, F).

Since YTHDF2 and YTHDF3 are known regulators of RNA stability^21^, we analyzed their correlation with YAP1 using TCGA datasets. A strong correlation was observed between YTHDF3 and YAP1 expression (Figure 5G), suggesting YTHDF3 may participate in YAP1 m6A modification. To validate this, we established YTHDF3-knockdown PANC-1 and BxPC-3 PDAC cell lines. YTHDF3 depletion led to significant downregulation of YAP1 expression (Figure 5H and S2G).

Furthermore, RNA pull-down assays confirmed direct binding between YTHDF3 and YAP1 mRNA (Figure 5I). RIP-qPCR also demonstrated enriched binding of YTHDF3 to YAP1 mRNA (Figure 5J). Finally, YTHDF3 knockdown substantially decreased the stability of YAP1 mRNA in both PANC-1 and BxPC-3 cells (Figure 5K, L).

Collectively, these findings demonstrate that METTL14 promotes YAP1 expression by enhancing the m6A methylation of YAP1 mRNA, which in turn recruits YTHDF3 to stabilize the transcript, thereby revealing a functional and mechanistic axis critical for YAP1 regulation in PDAC cells.

### Mechanotransduction-mediated nuclear translocation of YAP1 promotes METTL14 expression, forming a positive feedback loop

**Figure 6:**
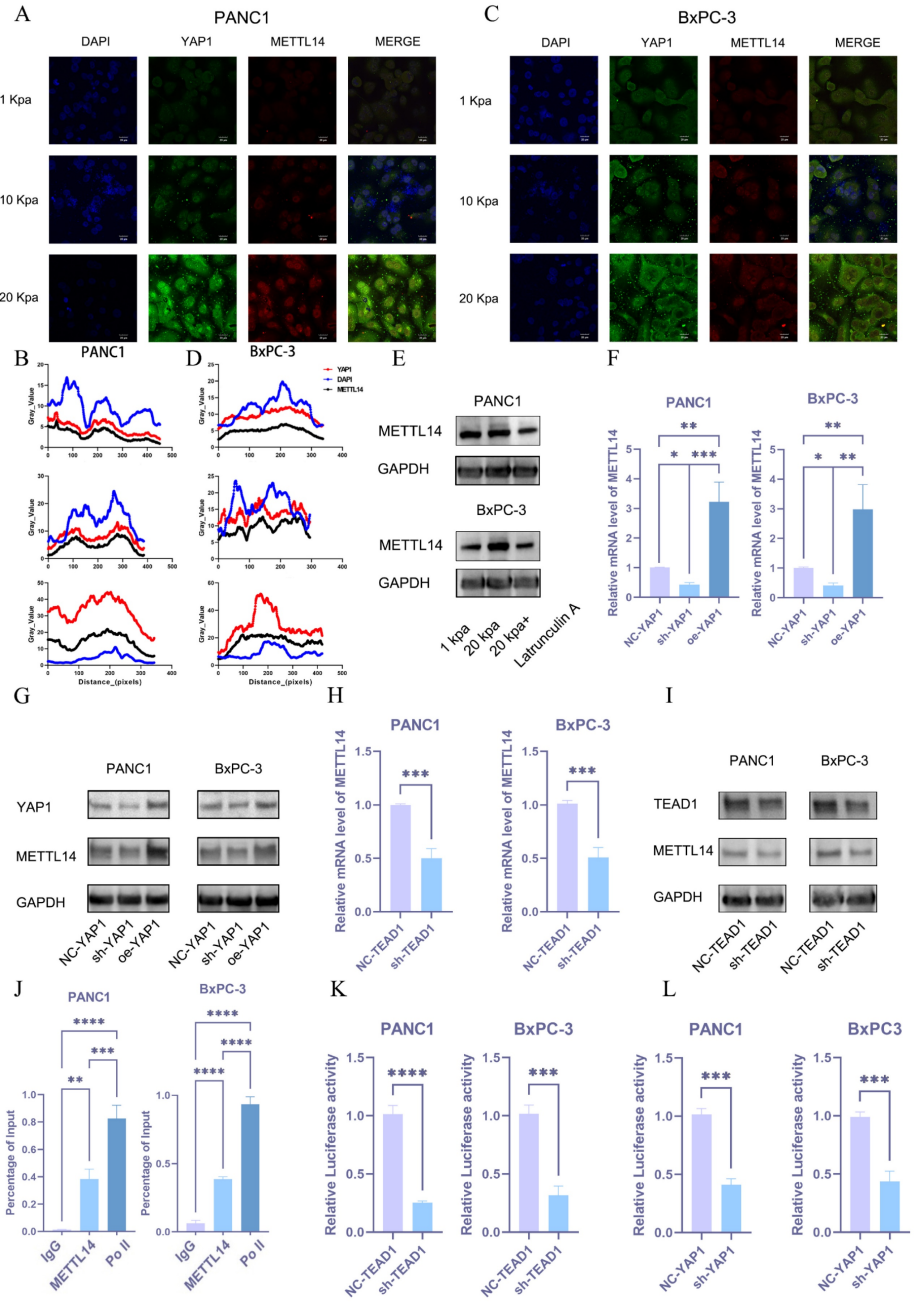
** Mechanotransduction-mediated nuclear translocation of YAP1 promotes METTL14 expression, forming a positive feedback loop.** (A-D) Expression levels of YAP1 and METTL14 in PANC1 (A) and BxPC-3 (C) cells cultured under varying stiffness conditions, as assessed via confocal microscopy; Immunofluorescence analysis of PANC1 (B) and BxPC-3 cells (D). (E) Protein expression levels of METTL14 under a 20 kPa stiffness culture environment and following the addition of Latrunculin were evaluated via western blot; n = 3. (F-G) Quantification of METTL14 mRNA (F) and protein (G) expression levels under YAP1 knockdown or overexpression, as determined via PCR and western blot; n = 3. (H-I) Alterations in METTL14 expression levels, as measured via PCR (H) and western blot (I), following TEAD1 knockdown; n = 3. (J) Chromatin immunoprecipitation (ChIP) assay results encompassing TEAD1 and the predicted fragments of the METTL14 promoter; n = 3. (K, L) Variations in the activity of a luciferase reporter controlled by the METTL14 promoter after TEAD1 (K) and YAP1 (L) knockdown; n = 3. Data are presented as mean ± standard of error (SD). Statistical significance was determined using ANOVA with post-hoc Tukey multiple comparison, * P < 0.05, ** P < 0.01, *** *P* < 0.0001, n.s.: not significant.

Then, we aimed to understand how METTL14 is upregulated during the process of PDAC matrix stiffening. YAP1 may be involved in the process as a sensor and mediator of mechanical signals within the cellular microenvironment. To validate this hypothesis, we performed immunofluorescence staining on pancreatic cancer cell lines grown under different stiffness conditions. An increase in stiffness significantly promoted the nuclear translocation of YAP1 and METTL14 (Figure 6A-D). Western blot analysis also confirmed that substrate stiffness promotes YAP expression and nuclear translocation. (Figure S2H). We also found that m6A methylation depends on cytoskeletal integrity and cellular tension, as the disruption of actin filaments or microtubules with latrunculin A significantly downregulated METTL14 (Figure 6E and S2I). We hypothesized that matrix stiffness promotes METTL14 expression via the YAP1 mechanotransduction pathway. Utilizing PCR and western blot analyses, we examined the expression of METTL14 in BxPC-3 cells with YAP1 knockdown or overexpression. These results indicated that METTL14 was significantly downregulated following YAP1 knockdown and markedly upregulated upon overexpression (Figure 6F, G and S2J). YAP1 is a transcriptional co-activator that lacks a DNA-binding domain, necessitating its interaction with other transcription factors (primarily the TEAD family) to regulate gene expression^22^. We analyzed the correlation between TEAD1-4 and METTL14 expression in PDAC to find a strong correlation between METTL14 and TEAD1 (Figure S3A). We hypothesized that YAP1 might regulate METTL14 expression through TEAD1. We used shRNA to knockdown TEAD1 in PANC1 and BxPC-3 cells followed by PCR and western blotting to detect METTL14 expression. METTL14 was significantly downregulated under TEAD1 knockdown (Figure 6H, I and S3B). We then used the Jaspar database to predict the TEAD1 binding site within the promoter region of METTL14 and designed primers for ChIP-qPCR based on the predicted sites. This experiment confirmed that TEAD1 directly binds to the METTL14 promoter region (Figure 6J). To validate the regulatory role of TEAD1 in METTL14 expression, we constructed a reporter gene plasmid containing the METTL14 promoter sequence and co-transfected it with the control plasmid into PANC1 and BxPC-3 PDAC cells with TEAD1 knockdown (sh-TEAD1) or control (NC-TEAD1). TEAD1 knockdown significantly inhibited METTL14 promoter activity (Figure 6K). Similar results were obtained when these plasmids were co-transfected into PANC1 and BxPC-3 PDAC cells with YAP1 knockdown (sh-YAP1) or control (NC-YAP1) (Figure 6L). These results demonstrate that matrix stiffness activates the YAP1-TEAD1 axis, which in turn transcriptionally upregulates METTL14 expression.

### YAP1 mediates stiffness-induced mechanical memory and stemness via the CD166-LOXL2 axis

**Figure 7:**
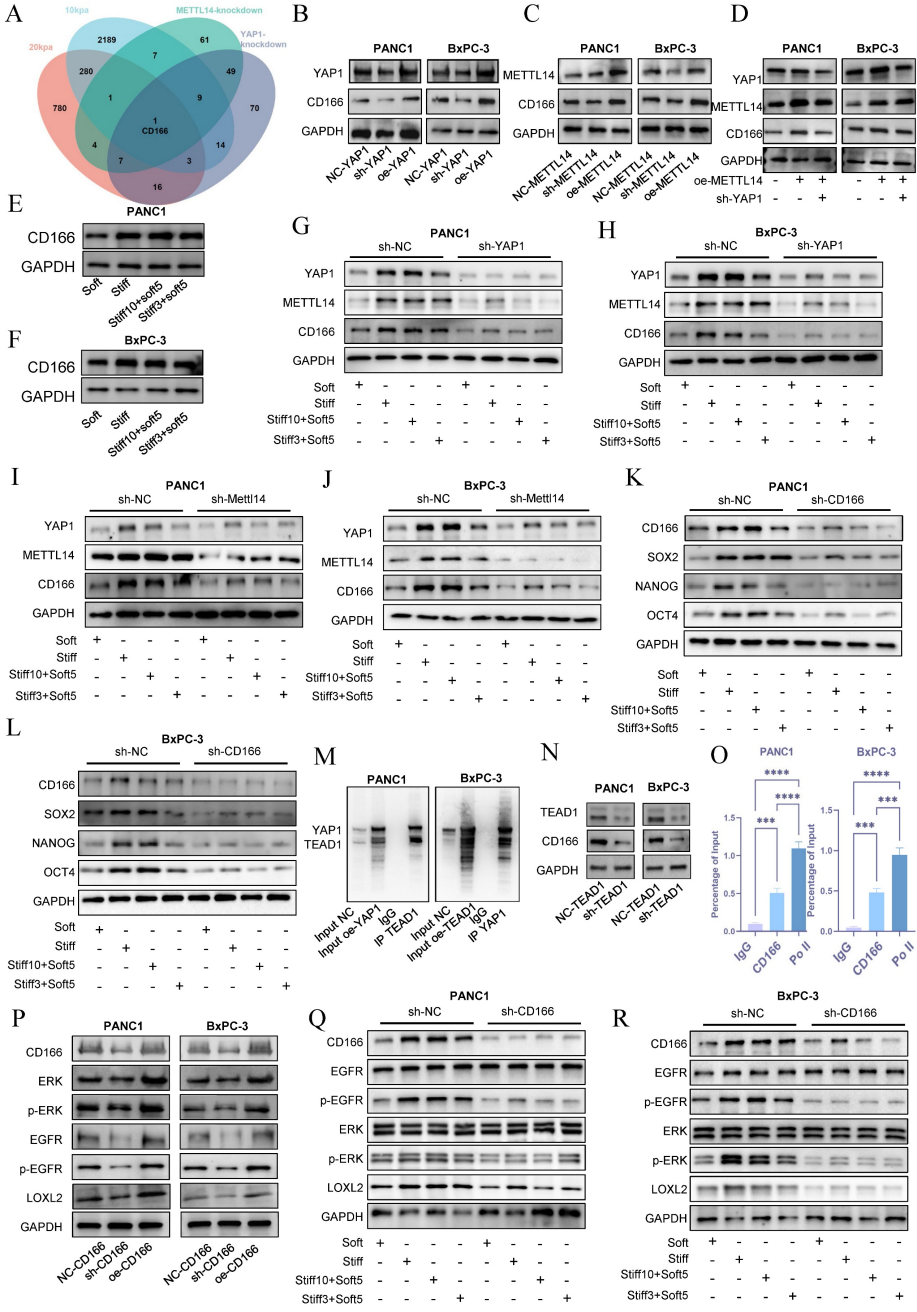
**YAP1 mediates stiffness-induced mechanical memory and stemness via the CD166-LOXL2 axis.** Venn diagram depicting the intersection of downregulated genes following METTL14 and YAP1 knockdown as well as of genes upregulated under 10 kPa and 20 kPa stiffness culture conditions. (B-C) Evaluation of CD166 expression through western blotting after YAP1 (B) and METTL14 (C) knockdown and overexpression; n = 3. (D) Rescue experiments confirmed the regulatory role of the METTL14-YAP1-CD166 axis; n = 3. (E-F) PANC-1 and BxPC-3 were cultured at 20 kPa for 10 days (to induce mechanical memory formation) or 3 days (without mechanical memory formation), followed by transfer to a 1 kPa culture condition for 5 days, and the expression level of CD166 was detected by western blotting; n = 3. (G-H) YAP1 was knocked out in PANC-1 and BxPC-3 cells first. Then cells were then cultured at 20 kPa for 10 days (to induce mechanical memory formation) or 3 days (without mechanical memory formation), followed by transfer to a 1 kPa culture condition for 5 days, and the expression level of CD166, METTL14 and YAP1 were detected by western blotting; n = 3. (I-J) METTL14 was knocked out in PANC-1 and BxPC-3 cells first. Then cells were then cultured at 20 kPa for 10 days (to induce mechanical memory formation) or 3 days (without mechanical memory formation), followed by transfer to a 1 kPa culture condition for 5 days, and the expression level of CD166, METTL14 and YAP1 were detected by western blotting; n = 3. (K-L) The expression level of CD166, SOX2, NANOG and OCT4 were detected by western blotting in PANC-1 and BxPC-3; n = 3. (M) Co-immunoprecipitation (Co-IP) results corroborated the interaction between TEAD1 and YAP1; n = 3. (N) Alterations in CD166 expression levels detected via western blot following TEAD1 knockdown; n = 3. (O) Chromatin immunoprecipitation (ChIP) assay results involving TEAD1 and predicted fragments of the CD166 promoter; n = 3. (P) Western blot analyses detailing alterations in the EGFR pathway and LOXL2 expression following CD166 knockdown or overexpression; n = 3. (Q-R) CD166 was knocked out in PANC-1 and BxPC-3 cells first. Then cells were then cultured at 20 kPa for 10 days (to induce mechanical memory formation) or 3 days (without mechanical memory formation), followed by transfer to a 1 kPa culture condition for 5 days, the regulatory effect of CD166 on LOXL2 during mechanical memory was detected by Western Blotting; n = 3. Data are presented as mean ± standard of error (SD). Statistical significance was determined using ANOVA with post-hoc Tukey multiple comparison, * P < 0.05, ** P < 0.01, *** *P* < 0.0001, n.s.: not significant.

To investigate the molecular basis by which the METTL14-YAP1 positive feedback circuit regulates tumor stromal stiffening and mechanical memory development, we established sh-YAP1 and oe-YAP1 PANC-1 and BxPC-3 cell lines via lentiviral transduction and performed transcriptomic sequencing in NC-YAP1 and sh-YAP1 BxPC-3 cells. Gene Ontology (GO) analysis indicated significant enrichment of extracellular matrix-related pathways upon YAP1 knockdown (Figure S3C), consistent with our earlier hypothesis. KEGG enrichment analysis revealed that downregulated genes were associated with Focal adhesion, ECM-receptor interaction, and Cell adhesion molecules (Figure S3D). By intersecting transcriptome data from cells cultured on 10 kPa and 20 kPa substrates with sh-YAP1 and sh-METTL14 conditions, we identified CD166 as the only gene significantly enriched across all four comparisons (Figure 7A).

Western blot analysis showed that YAP1 knockdown markedly suppressed CD166 protein expression, while YAP1 overexpression enhanced it in both PANC-1 and BxPC-3 cells (Figure 7B and S4A). Similarly, METTL14 knockdown reduced and overexpression increased CD166 expression (Figure 7C and S4B). Importantly, YAP1 knockdown reversed METTL14 overexpression-induced CD166 upregulation (Figure 7D and S4C). Stiffness significantly upregulated CD166 protein expression and established mechanical memory (Fig. 7E-F and S4D), while disruption of the METTL14-YAP1 positive feedback axis potently reversed this mechano-adaptation (Fig. 7G-J and S4E-H). Intriguingly, CD166 exhibited enhanced mechanical memory propensity—culturing on rigid matrices (20 kPa) for merely 3 days sustained stable expression levels that persisted even after transfer to soft substrates (1 kPa) (Fig. 7E-J). Furthermore, as a canonical stemness marker, CD166 knockdown potently abrogated the stemness-enhancing effects induced by both matrix rigidity and mechanical memory (Figure 7K-L and S4I-J). Inhibition of the YAP1-METTL14 loop produced similar effects (Figure S5A-D), supporting the role of this feedback circuit in maintaining stemness and mechanical memory via CD166.

To explore how METTL14-YAP1 axis regulates CD166, we considered that YAP1 acts as a transcriptional co-activator requiring DNA-binding partners such as TEAD family proteins. Correlation analysis in PDAC indicated a strong association between CD166 and TEAD1 (Figure S5E). Co-immunoprecipitation confirmed YAP1-TEAD1 interaction (Figure 7M). TEAD1 knockdown reduced CD166 expression (Figure 7N and S5F), and ChIP assays demonstrated TEAD1 binding to the CD166 promoter (Figure 7O), indicating YAP1 regulates CD166 through TEAD1.

Matrix cross-linking represents a critical determinant of matrix stiffness, with LOXL2 functioning as a key driver of this cross-linking process^23^. Analysis using the GEPIA2 database revealed a statistically significant positive correlation between the expression of CD166 (ALCAM) and LOXL2, which underscores their functional cooperation in the development of matrix stiffness (Figure S5G). Enrichment analysis of differentially expressed genes revealed that elevated matrix stiffness induces upregulation of the EGFR tyrosine kinase inhibitor resistance pathway^23^ (Figure S6A). Furthermore, accumulating evidence indicates that CD166 enhances tumor stemness and malignant progression through activation of the EGFR pathway. Based on these observations, we hypothesized that CD166 modulates LOXL2 expression via EGFR-mediated signaling, thereby promoting matrix stiffening. CD166 knockdown decreased EGFR signaling and LOXL2 expression, while its overexpression had the opposite effect (Figure 7P and S6B). Tumor cell with mechanical memory can maintain the activation of the CD166-EGFR-LOXL2 axis, promoting stromal stiffening—a effect reversed by CD166 inhibition (Figure 7Q, R and S6C-E). EGFR inhibitor treatment mitigated CD166-induced LOXL2 upregulation (Figure S6F). Additionally, CD166 knockdown resulted in smoother cell surfaces and reduced collagen fibers (Figure S6G), confirming its role in fibrotic cross-Linking via LOXL2. In conclusion, these results demonstrate that the CD166-LOXL2 axis acts downstream of METTL14-YAP1 axis in regulating PDAC stiffness and mechanical memory. Furthermore, tumor cells with mechanical memory remodel the extracellular matrix at metastatic sites, facilitating a permissive niche for growth.

### CD166 promotes PDAC tumor stiffness through LOXL2

**Figure 8:**
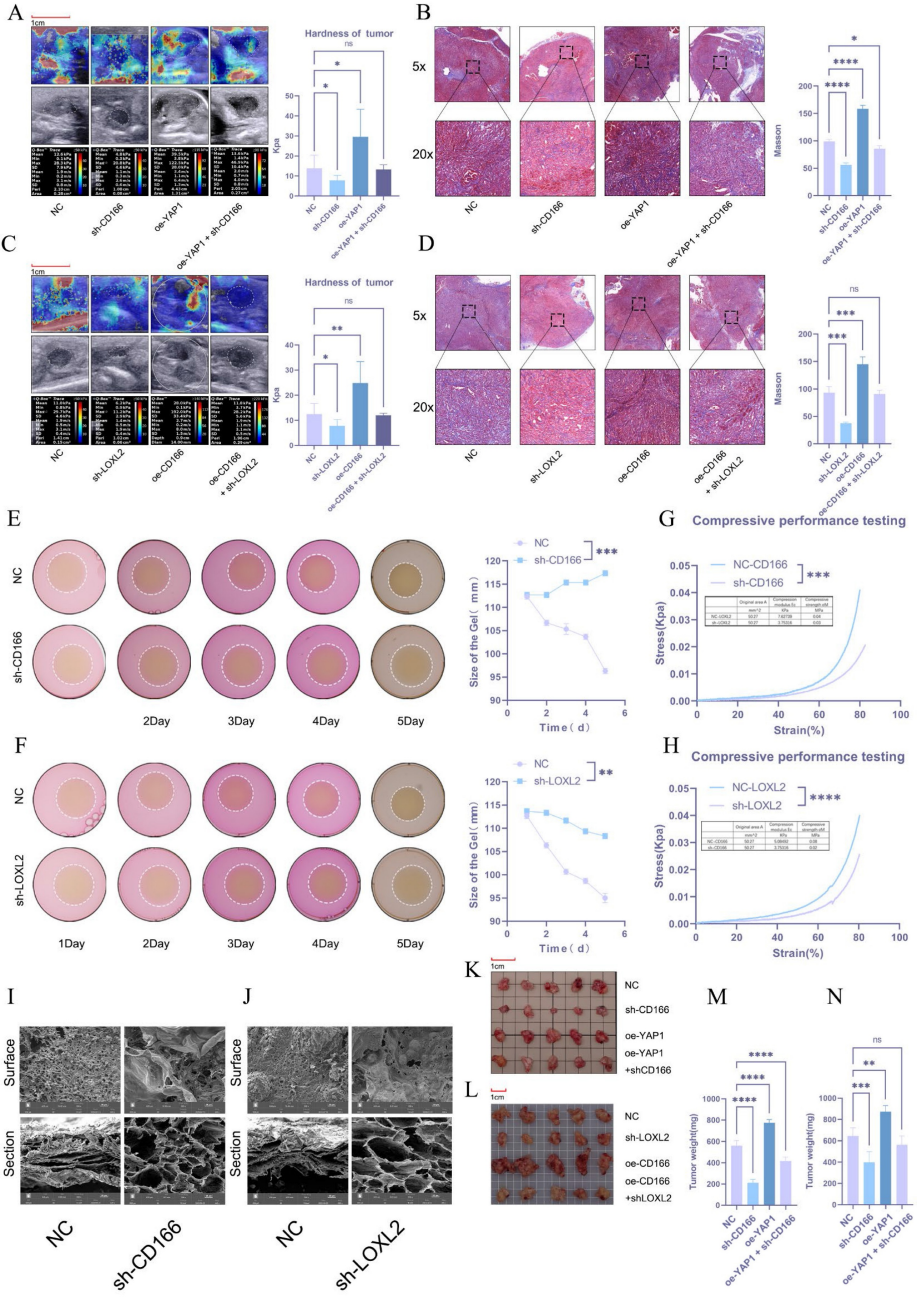
**CD166 promotes PDAC tumor stiffness through LOXL2.** (A, C) Results of elastography to test the tumor stiffness in the *in situ* PDAC tumor model. (B, D) Results of Masson's trichrome staining to detect the degree of fibrosis of the *in situ* tumor model of PDAC. (E-F) Results of the matrix cross-linking experiment of BxPC-3 PDAC cells after knocking down CD166 (E) and LOXL2 (F). (G-H) An electronic universal testing machine is used to measure the matrix stiffness of collagen fibers after 3D culture. (I-J) Morphological changes in the type I collagen fiber matrix observed under an electron microscope after knocking down CD166 (I) and LOXL2 (J). (K-L) Tumor size in the *in situ* tumor model mice. (M-N) Tumor weight in the *in situ* tumor model mice.

The regulatory effect of CD166-LOLX2 on stroma stiffness was further validated in tumor models established using CD166 knockdown or CD166-overexpressing KPC cells. Ultrasound elastography first verified that CD166 knockdown significantly reversed matrix stiffening caused by YAP1 overexpression, which was in line with Masson's trichrome staining (Figure 8A, B). Similarly, LOXL2 knockdown reduced stroma stiffness and abolished the effect of CD166 overexpression (Figure 8C, D). The effect of LOXL2 on matrix cross-linking was studied via 3D cell culture in a type I collagen matrix. PDAC cells with CD166 or LOXL2 knockdown significantly reduced matrix contractibility (Figure 8E, F). To validate whether cross-linking affects matrix stiffness, we utilized an electronic universal testing machine to assess the stiffness of NC-CD166 and sh-CD166 with collagen fibers after 3D culture. Knocking down CD166 and LOXL2 significantly reduced matrix stiffness (Figure 8G, H). The lyophilized collagen matrix also exhibited large pore sizes and loose structure under an electron microscope (Figure 8I, J). Notably, CD166 knockdown resulted in a smooth cell surface and a significant reduction in collagen fibers, which was in line with our results (Figure S1I). These results confirmed that CD166 affects PDAC fibrotic cross-linking by regulating LOXL2 to promote matrix stiffening. The in-situ tumor model of C57 mice showed that CD166 knockdown significantly inhibited tumor growth, while suppressing the tumor progression triggered by YAP1 overexpression (Figure 8K). In addition, knocking down CD166 and LOXL2 significantly inhibited orthotopic tumor growth in mice. More importantly, CD166 knockdown suppressed the promoting effect of YAP1 overexpression on pancreatic cancer growth, while LOXL2 knockdown suppressed the promoting effect of CD166 overexpression on pancreatic cancer growth (Figure 8K, L, M, N).

### Mechanical memory driven by substrate stiffness maintains stemness and confers gemcitabine resistance in pancreatic cancer

**Figure 9:**
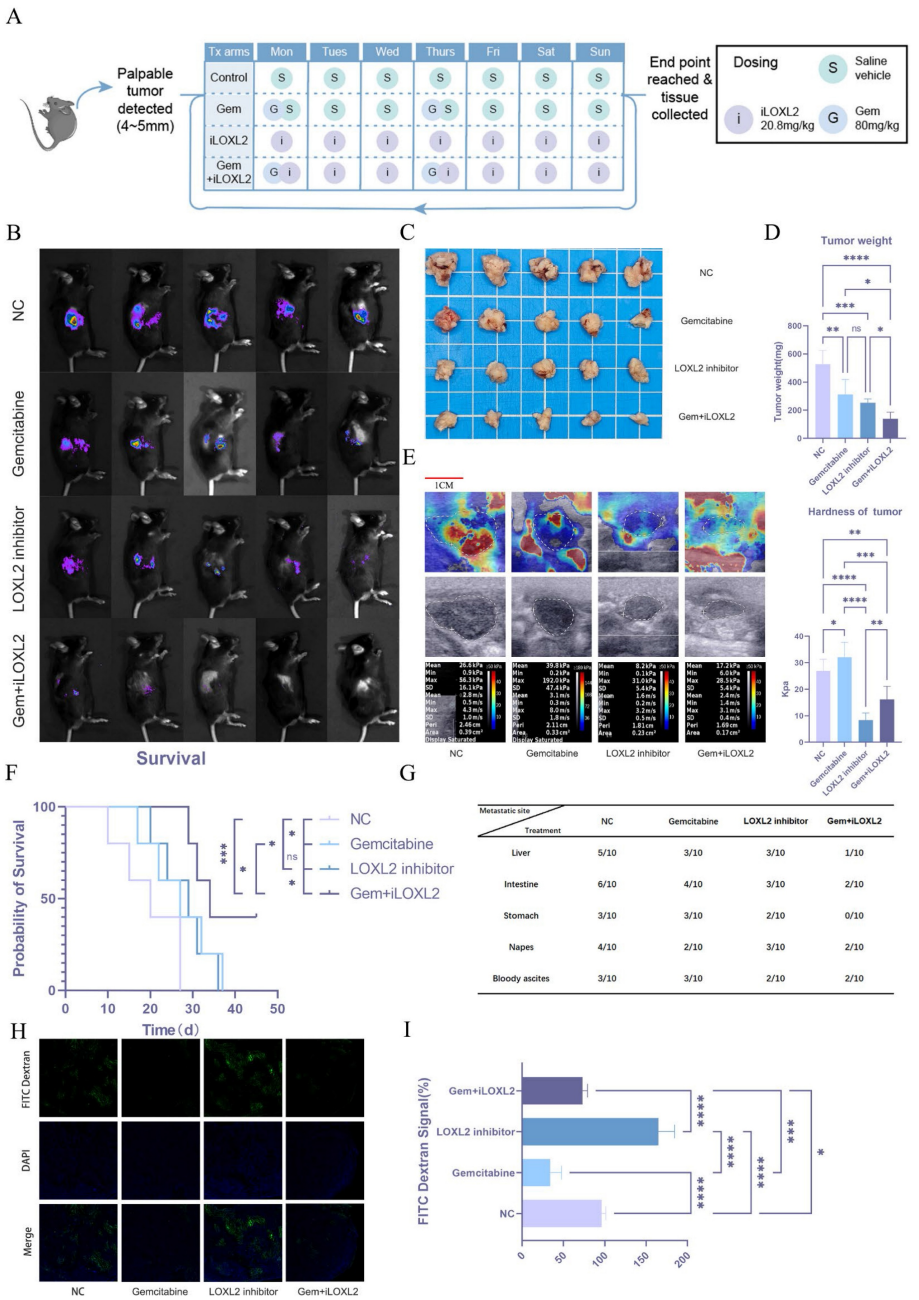
** Mechanical memory driven by substrate stiffness maintains stemness and confers gemcitabine resistance in pancreatic cancer.** Schematic diagram of drug administration in the animal model. *In vivo* imaging results in mice; n = 5. (C-D) Tumor volume (C) and weight (D) results in mice; n = 5. (E) Elastic ultrasound stiffness detection results in mice. (F) Prognostic data of mice in different treatment groups. (G) Data on distant metastasis in mice from different treatment groups. (H-I) Immunofluorescence of intratumoral infusion with FITC-dextran (H) and quantitative analysis (I). Data are presented as mean ± standard of error (SD). Statistical significance was determined using ANOVA with post-hoc Tukey multiple comparison, * P < 0.05, ** P < 0.01, *** *P* < 0.0001, n.s.: not significant.

Stiffness and mechanical memory play essential roles in the establishment and maintenance of tumor stemness. Tumor cells with stem-like properties possess self-renewal and multi-lineage differentiation capabilities, which drive tumor heterogeneity and serve as key factors in tumor recurrence, metastasis, and therapy resistance. We hypothesized that increased matrix stiffness in pancreatic cancer contributes to gemcitabine resistance, thereby negatively impacting patient prognosis. Given the critical role of LOXL2 in collagen deposition and matrix remodeling, we aimed to modulate tumor matrix stiffness by targeted inhibition of LOXL2. To test this hypothesis, we established an orthotopic mouse model of pancreatic cancer and treated the mice with normal saline (NC), gemcitabine (Gem), a LOXL2 inhibitor (iLOXL2), or a combination of gemcitabine and the LOXL2 inhibitor (Gem + iLOXL2) (Figure 9A). In vivo imaging results showed that both gemcitabine and the LOXL2 inhibitor could inhibit tumor growth, but the effect was not significant. However, combination of the two significantly inhibited tumor progression (Figure 9B). The same effect was observed for tumor volume and weight in mice (Figure 9C, D). Further, combined treatment significantly extended the survival of mice (Figure 9F) and reduced the metastatic potential of pancreatic cancer (Figure 9G). Using elastic ultrasound, we found that gemcitabine treatment significantly increased tumor stiffness, while the LOXL2 inhibitor decreased it and could mitigate the gemcitabine-induced increase in stiffness (Figure 9E). We speculated the increased tumor fibrosis after gemcitabine treatment leads to greater stiffness, which reduces gemcitabine tumor perfusion. To verify this hypothesis, we injected mice with FITC-labeled dextran (MW10000, to simulate gemcitabine) via the tail vein 4 h before euthanasia. Tumor drug perfusion was then assessed in frozen sections. Drug perfusion significantly decreased following gemcitabine treatment. In contrast, treatment targeting tumor matrix stiffness significantly enhanced drug perfusion and mitigated the gemcitabine-induced reduction in perfusion (Figure 9H, I).

## Discussion

Tumors typically exhibit significantly increased stiffness compared to normal tissues, and exogenous tissue stiffening has been shown to actively promote tumor initiation and progression. Consequently, ECM stiffness has emerged as a critical biomechanical parameter in tumorigenesis, influencing multiple facets of cancer cell behavior^24^. For example, ECM stiffness has been demonstrated to enhance the proliferation and migration of glioma cells, correlate with poor prognosis in neuroblastoma, and regulate stem cell self-renewal and differentiation processes^25^,^26^. Using ultrasound elastography, we observed that elevated tumor stiffness in clinical PDAC cases was associated with advanced nodal (N) and distant metastasis (M) stages, as well as shorter overall survival. Furthermore, in situ tumor models in KPC mice revealed that matrix stiffness increases progressively during tumor development, reinforcing the role of biomechanical cues in disease aggressiveness and supporting its potential value as a prognostic biomarker.

Sustained mechanical stimulation within a high-stiffness extracellular matrix induces the formation of a mechanical memory in tumor cells, driven by durable epigenetic and transcriptional reprogramming. This memory endows cancer cells with persistently aggressive pro-metastatic traits—such as enhanced migratory capacity, stem-like properties, and chemoresistance—that persist even after dissemination into softer secondary sites^8^. Consequently, these adaptations significantly accelerate metastatic colonization and malignant evolution^7^. Our findings establish that mechanical memory promotes malignant progression through the sustained maintenance of tumor stemness. Moreover, this process initiates a vicious, self-reinforcing cycle: the establishment of mechanical memory further stimulates ECM stiffening, which in turn exacerbates the rigidity of the tumor microenvironment. This feedforward mechano-pathological loop critically contributes to accelerated disease progression and worse outcomes in pancreatic cancer.

Transcriptomic sequencing revealed differential gene expression profiles in tumor cells under varying stiffness conditions, with significant enrichment in RNA modification- related pathways. Elevated levels of m6A methylation were detected in response to increasing matrix stiffness, indicating a strong correlation between m6A abundance and ECM rigidity^17^,^27^. Furthermore, m6A-mediated epigenetic modifications play a critical role in the establishment and maintenance of mechanical memory in tumor cells. By comparing the expression levels of various m6A writers and erasers, we found that METTL14 was significantly upregulated in tumor cells under high-stiffness conditions. Tissue microarray analysis indicated that high METTL14 levels were associated with poor prognosis in patients with PDAC. Immunofluorescence and Masson staining of clinical specimens showed a positive correlation between METTL14 fluorescence intensity and Masson staining intensity. Mechanical memory induces METTL14-dependent epigenetic changes. We therefore hypothesize that elevated METTL14 expression underlies increased matrix stiffness in PDAC. Critically, once established, tumor mechanical memory perpetuates high METTL14 levels, creating a self-reinforcing loop that exacerbates matrix stiffening and ultimately culminates in poor prognosis. Animal experiments confirmed this hypothesis, as METTL14 overexpression promoted in situ tumor growth and increased tumor stiffness and fibrosis, significantly shortening the overall survival.

YAP1 serves as a core transcriptional co-activator and key downstream effector of the Hippo signaling pathway, playing a pivotal role in regulating essential biological processes such as proliferation, differentiation, and apoptosis^11^,^12^,^28^,^29^. It also functions as a critical mechanosensor, translating extracellular biomechanical signals—especially elevated matrix stiffness—into transcriptional programs that drive malignant progression^30^,^31^. Moreover, YAP1 operates as an intracellular mechanical regulator essential for the acquisition and sustainment of mechanical memory in cancer cells^12^. Our results demonstrate that METTL14 enhances the translation efficiency of YAP1 mRNA in an m6A-dependent manner through the reader protein YTHDF3, thereby post-transcriptionally regulating YAP1 expression. This finding is consistent with prior reports highlighting the role of YAP1 in cancer-associated fibroblasts, where it promotes matrix stiffening and enhances invasive behavior. In its unphosphorylated state, YAP1 translocates into the nucleus and interacts with transcription factors of the TEAD family, activating the expression of genes that drive proliferation, migration, invasion, and apoptosis resistance^22^,^29^. For example, in breast cancer and melanoma, YAP1-TEAD1 cooperativity has been explicitly linked to tumor progression and metastasis. Together, these insights establish the METTL14-m6A-YTHDF3 axis as a pivotal upstream regulator of YAP1 expression and function, thereby bridging epitranscriptomic mechanisms with biomechanical signaling and malignant reprogramming in cancer.

Our findings position METTL14 as a critical regulator promoting stromal stiffening in PDAC, while also indicating that increased matrix stiffness upregulates METTL14 expression and modulates malignant behavior. Mechanistically, METTL14 facilitates YAP1 expression by enhancing the m6A-dependent translational efficiency of YAP1 mRNA, a process mediated by the m6A reader protein YTHDF3. Subsequently, YAP1 activation promotes METTL14 expression via the YAP1-TEAD1 transcriptional circuit, forming a self-reinforcing positive feedback loop that sustains mechanical memory in PDAC. Furthermore, the upregulated METTL14-YAP1 axis promotes LOXL2 transcription, resulting in enhanced collagen deposition and cross-linking that exacerbate stromal stiffness and accelerate disease progression. Importantly, our findings challenge the prevailing view that pancreatic tumor desmoplasia is predominantly driven by fibroblasts; instead, we demonstrate that pancreatic cancer cells themselves actively contribute to fibrosis and mechanical remodeling of the tumor microenvironment.

The LOX family of proteins are key regulators of collagen assembly and are frequently dysregulated in various solid tumors. Overactivation of the LOX family in the tumor microenvironment leads to excessive cross-linking of the extracellular matrix (ECM), increasing its stiffness and further promoting malignant progression^32^. Members of this family exhibit specific expression patterns across different cancer types. Studies have shown that LOX can drive the progression of multiple cancers, including colorectal, ovarian, lung, gastric, and renal cell carcinoma. Moreover, LOXL2 has been widely demonstrated to possess cancer-promoting functions, contributing to the development of colorectal, gastric, esophageal squamous cell, cholangiocarcinoma, hepatocellular, non-small cell lung, and renal cell cancers^33^.

Beyond the preclinical evidence, the clinical translational potential of targeting LOXL2 is underscored by ongoing investigative efforts. While earlier broad-spectrum lysyl oxidase inhibitors like β-aminopropionitrile demonstrated proof-of-concept, subsequent development has focused on specific neutralizing antibodies. Simtuzumab, a humanized monoclonal antibody against LOXL2, was evaluated in Phase II trials for idiopathic pulmonary fibrosis and advanced liver fibrosis but did not meet its primary endpoints, highlighting the challenges of monotherapy in late-stage fibrotic diseases^34^. Meanwhile, clinical studies have shown that the combination of sorafenib with simtuzumab did not result in significant abnormal toxicity and was associated with an improvement in median overall survival^35^. These clinical endeavors validate LOXL2 as a pharmacologically tractable target. We also showed that elevated stromal stiffness restricts intratumoral gemcitabine perfusion, fostering chemoresistance. Inhibition of LOXL2 mitigates fibrosis and restores gemcitabine efficacy, highlighting a promising therapeutic strategy to disrupt this mechanopathological cycle. The founding of this axis not only enhances our understanding of how the mechanical microenvironment regulates the formation of mechanical memory in cancer and accelerates malignant progression, but also provides novel therapeutic strategies targeting the mechanosignaling-epitranscriptional network, holding significant translational relevance.

## Supplementary Material

Supplementary figures.
