# Supplementary material for: Interaction between m6A and YAP1 mechanotransduction pathways is essential for mechanical memory and matrix remodeling in pancreatic cancer
Source: Int J Biol Sci. 2026 Feb 4;22(5):2302–23. doi: 10.7150/ijbs.125330 (PMC12965146; doi:10.7150/ijbs.125330)
Supplement: Supplementary file 1 — Supplementary figures. [file ijbsv22p2302s1.pdf]

Supplementary figures and figure legends

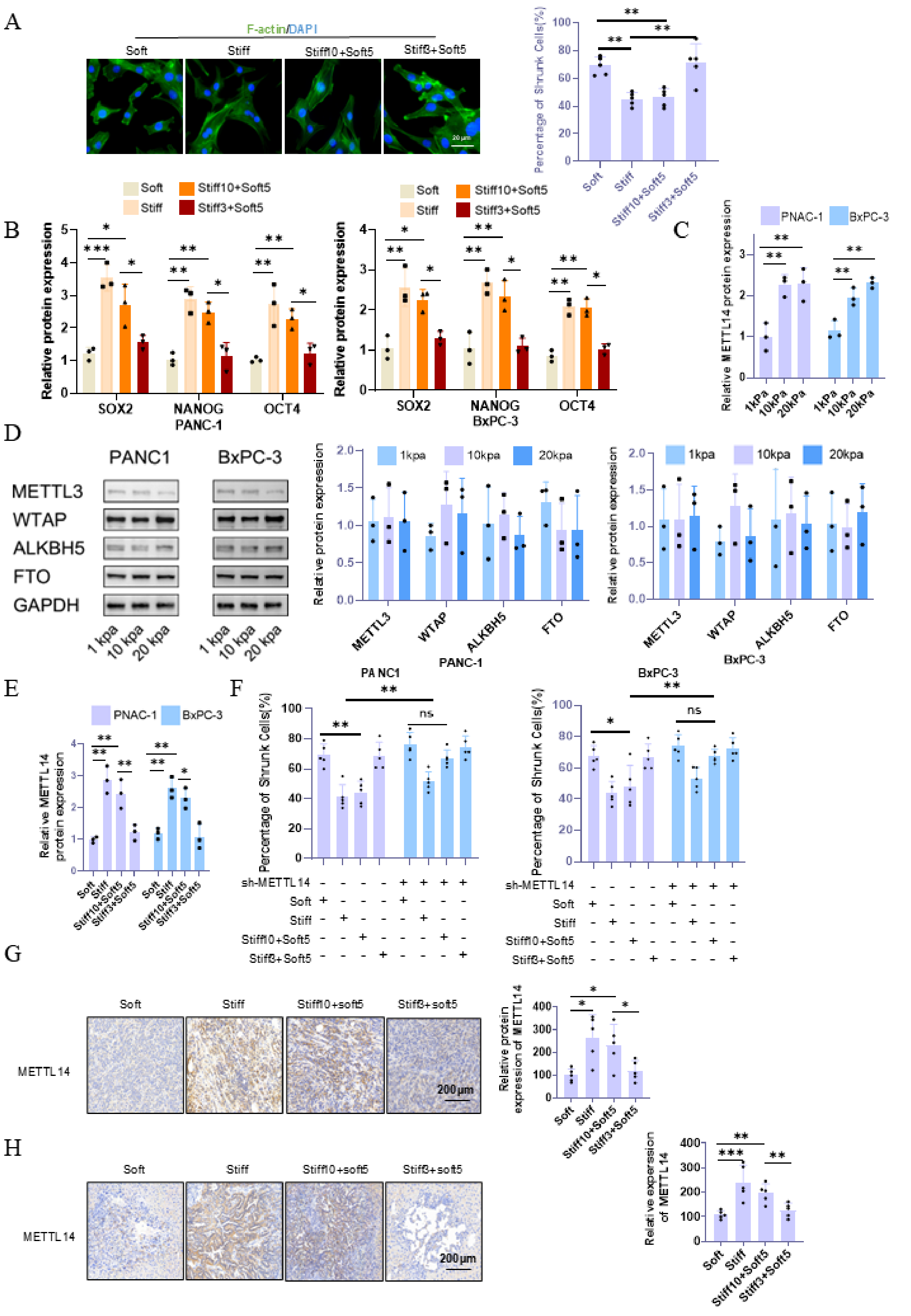

Fig. S1 (A) KPC cells were first cultured at 20 kPa for 10 days (to induce mechanical

memory formation) or 3 days (without mechanical memory formation). Subsequently, the cells were transferred to a 1 kPa culture environment for 5 days. F-actin immunofluorescence staining was performed to observe tumor cytoskeletal rearrangement to assess the role of mechanical memory in tumor cytoskeletal remodeling.

(B) Statistical graph of FIG2K. The PANC-1 and BxPC-3 were cultured at 20 kPa for 10 days (to induce mechanical memory formation) or 3 days (without mechanical memory formation), followed by transfer to a 1 kPa culture condition for 5 days, and the protein expression levels of CD166, METTL14 and YAP1 were subsequently quantified.

(C) Statistical graph of FIG3E. The PANC-1 and BxPC-3 were cultured under varying matrix stiffness conditions and the protein expression levels of METTL14 was subsequently quantified.

(D) Western blot analysis of m6A-related molecular expression levels in pancreatic cancer PANC1 and BxPC-3 cells cultured under different hardness conditions.

(E) To evaluate the role of tumor mechanical memory in maintaining METTL14 expression, PANC-1 and BxPC-3 cells were first cultured at 20 kPa for 10 days (to induce mechanical memory formation) or 3 days (without mechanical memory formation). And the protein expression levels of METTL14 was subsequently quantified.

(F) Statistical graph of F-actin staining in Fig. 2G.

(G-H) Immunohistochemical staining was employed to explore the role of mechanical memory in promoting METTL14 expression in the subcutaneous tumor model (G) and liver metastasis model (H).

Data are presented as mean ± standard of error (SD). Statistical significance was determined using ANOVA with post-hoc Tukey multiple comparison, * $P < 0.05$, ** $P < 0.01$, *** $P < 0.0001$, n.s.: not significant.

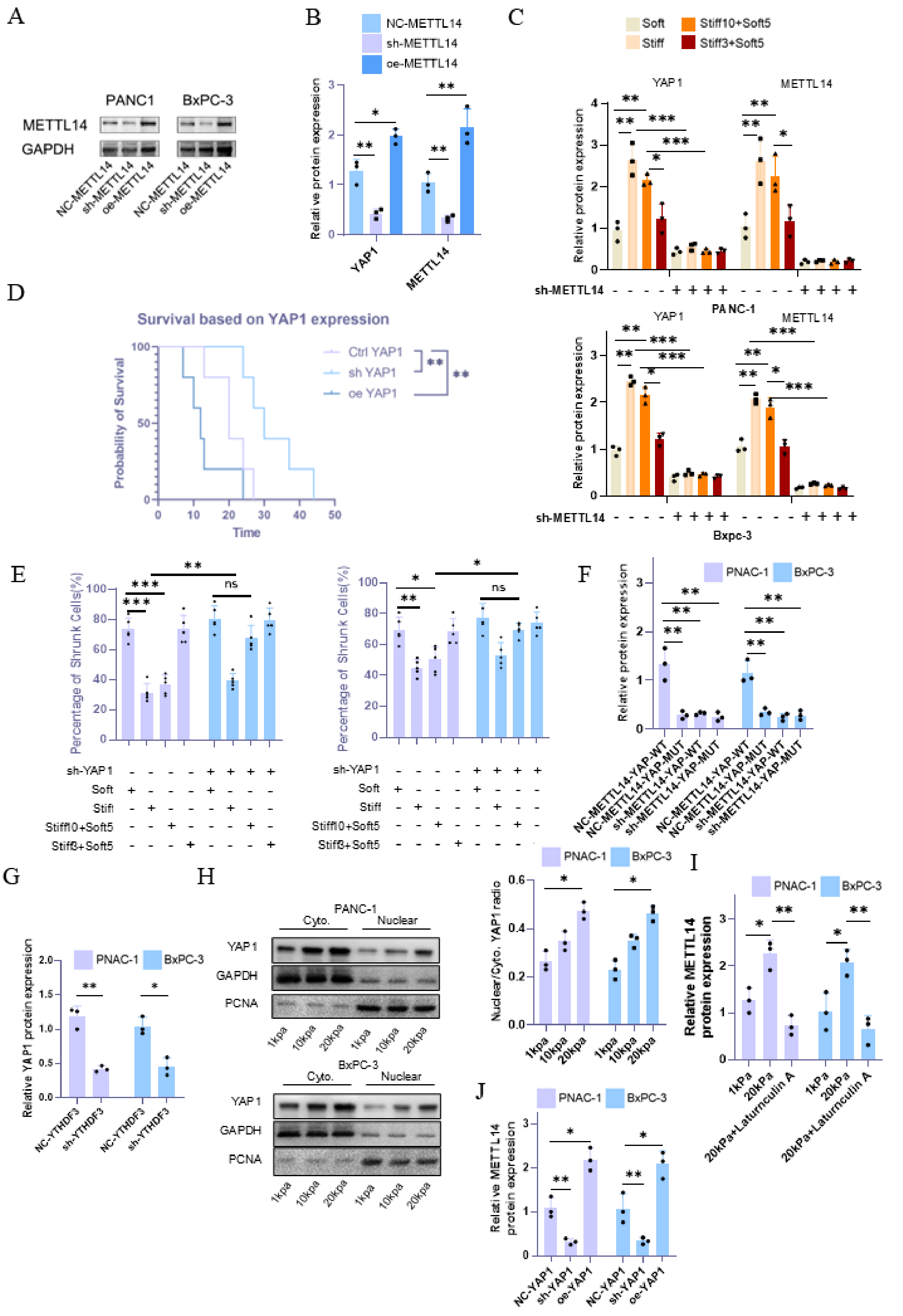

Fig. S2

(A)Western blot validation of METTL14 knockdown and overexpression efficiency in pancreatic cancer PANC1 and BxPC-3 cells.

(B)Statistical graph of FIG4D. Western blot analysis of m6A-related molecular expression levels in pancreatic cancer PANC1 and BxPC-3 cells cultured under different hardness conditions. The protein expression levels of METTL14 and YAP1 were subsequently quantified.

(C)Statistical graph of FIG4E. METTL14 was knocked out in PANC-1 and BxPC-3 cells first. Then cells were then cultured at 20 kPa for 10 days (to induce mechanical memory formation) or 3 days (without mechanical memory formation), followed by transfer to a 1 kPa culture condition for 5 days， and The protein expression levels of METTL14 and YAP1 were subsequently quantified.

(D)Survival analysis of mice with NC, YAP1 overexpression, and YAP1 knockdown.

(E)Statistical graph of F-actin staining in Fig. 3L-M.

(F)Statistical graph of FIG5D. Western blot detection results of YAP1 expression after m6A site mutation. The protein expression levels of YAP1 was subsequently quantified.

(G)Statistical graph of FIG5H. Western blot detection results of YAP1 expression after YTHDF3 knockdown in PANC1 and BxPC-3 cells. The protein expression levels of YAP1 was subsequently quantified.

(H)Western blot detection results of the nuclear/cyto YAP1 expression radio after culturing under different stiffness conditions. The protein expression levels of YAP1 was subsequently quantified.

(I)Statistical graph of FIG6E. Protein expression levels of METTL14 under a 20 kPa stiffness culture environment and following the addition of Latrunculin were evaluated via western blot. The protein expression levels of METTL14 was subsequently quantified.

(J) Statistical graph of FIG6G. Quantification of METTL14 protein expression levels under YAP1 knockdown or overexpression and the protein expression levels of

METTL14 was subsequently quantified.

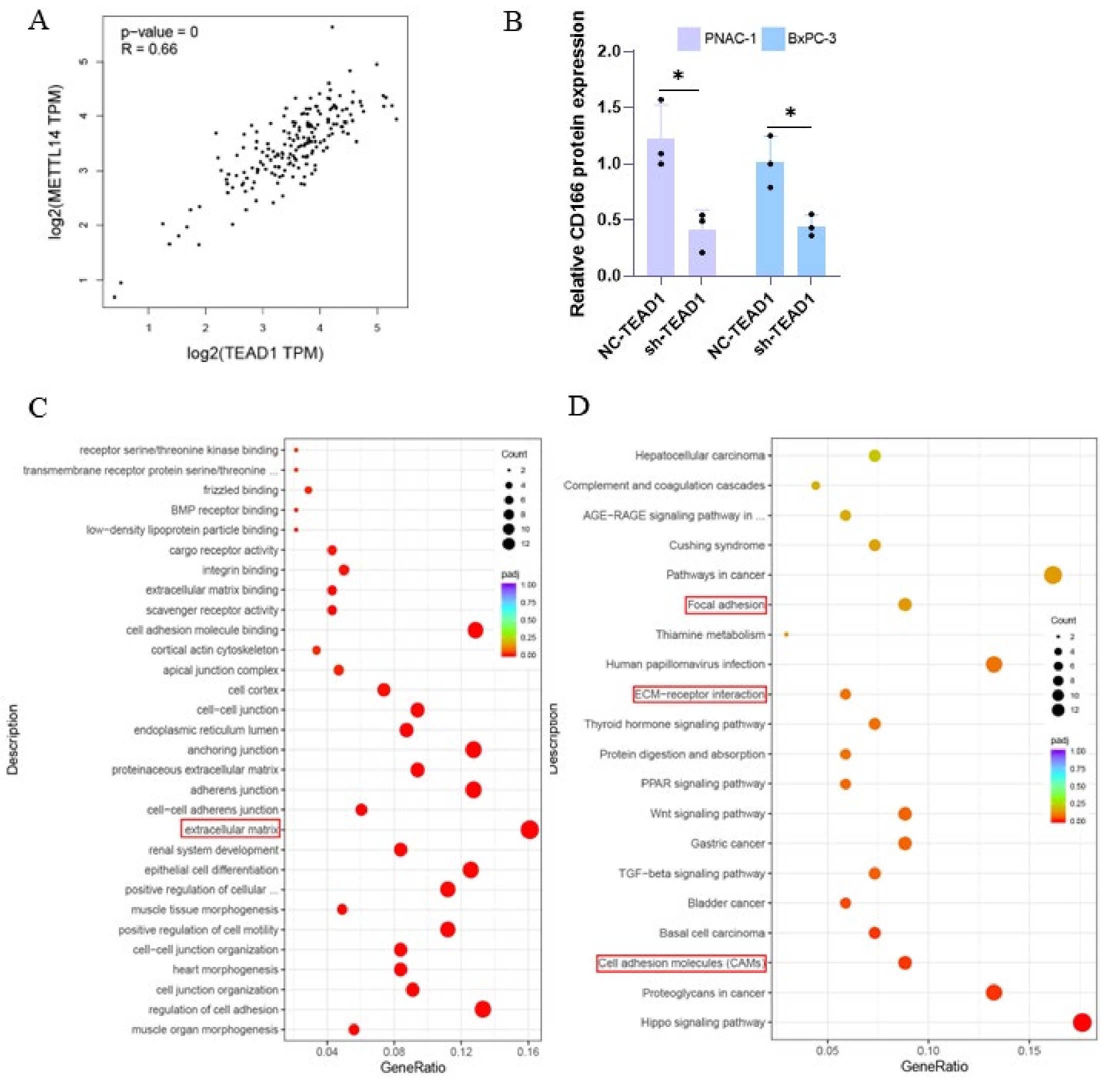

Fig. S3

(A)Analysis of the correlation between TEAD1 and METTL14 in the TCGA database.

(B)Statistical graph of FIG6I. Alterations in METTL14 expression levels, as measured via western blot, following TEAD1 knockdown. The protein expression levels of METTL14 was subsequently quantified.

(C) GO enrichment analysis of genes downregulated following YAP1 knockdown.

(D) KEGG enrichment analysis of genes downregulated following YAP1

knockdown.

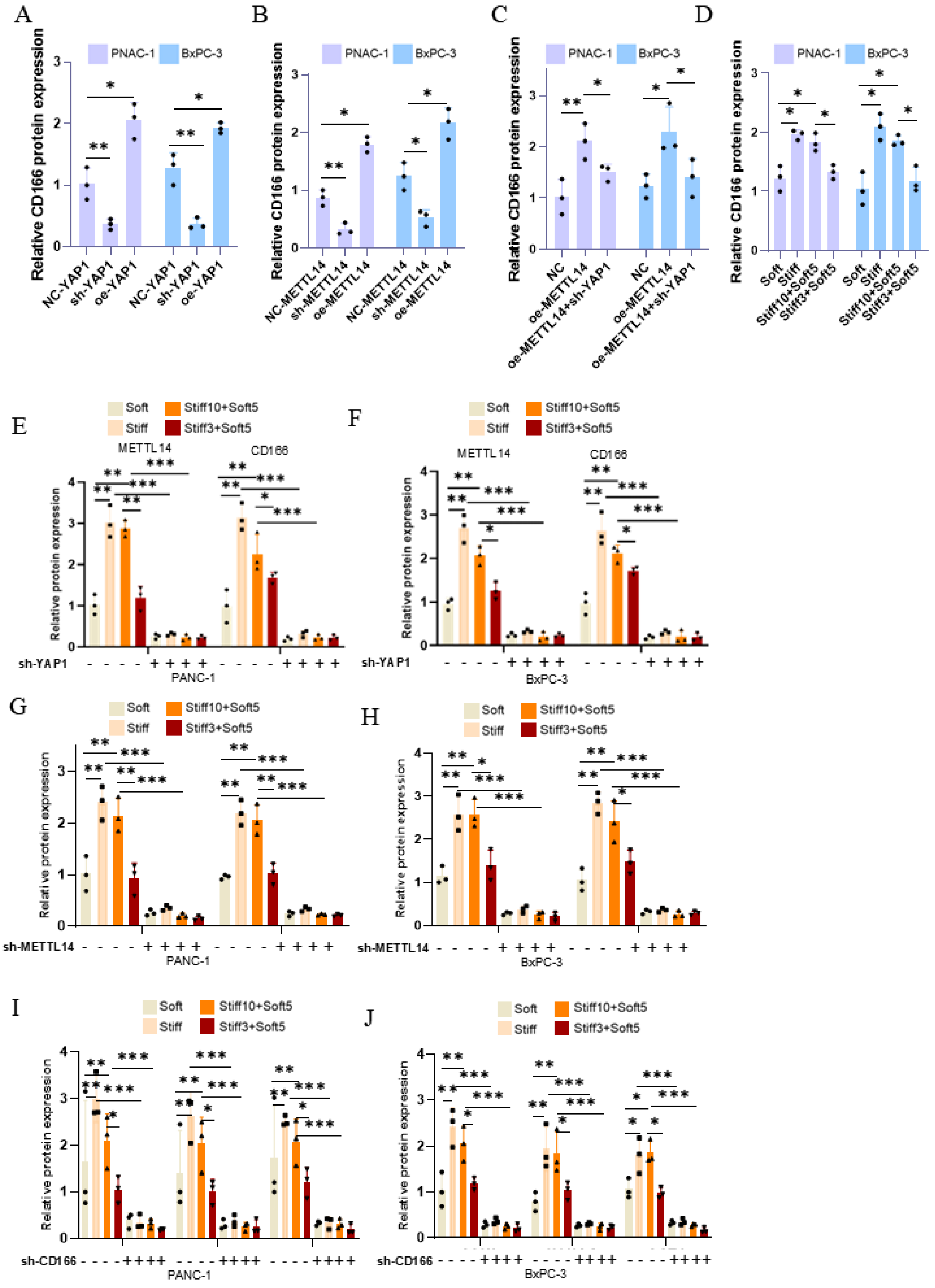
A
B
C
D
PNAC-1
BxPC-3
Relative CD166 protein expression
NC-YAP1
sh-YAP1
oe-YAP1
NC-METTL14
sh-METTL14
oe-METTL14
NC
oe-METTL14
oe-METTL14+sh-YAP1
Soft
Stiff
Stiff10+Soft5
Stiff3+Soft5
E
F
METTL14
CD166
Relative protein expression
sh-YAP1
PANC-1
BxPC-3
G
H
sh-METTL14
I
J
sh-CD166

Fig. S4

(A-B) Statistical graph of FIG7B and FIG7C. Evaluation of CD166 expression through western blotting after YAP1 (A) and METTL14 (B) knockdown and overexpression. The protein expression levels of CD166 was subsequently quantified.

(C) Statistical graph of FIG7D. Rescue experiments confirmed the regulatory role of the METTL14-YAP1-CD166 axis, and the protein expression levels of CD166 was subsequently quantified.

(D) Statistical graph of FIG7E and FIG7F. PANC-1 and BxPC-3 were cultured at 20 kPa for 10 days (to induce mechanical memory formation) or 3 days (without mechanical memory formation), followed by transfer to a 1 kPa culture condition for 5 days， and the expression level of CD166 was subsequently quantified.

(E-F) Statistical graph of FIG7G and FIG7H. YAP1 was knocked out in PANC-1 and BxPC-3 cells first. Then cells were then cultured at 20 kPa for 10 days (to induce mechanical memory formation) or 3 days (without mechanical memory formation), followed by transfer to a 1 kPa culture condition for 5 days, and the expression level of CD166 and METTL14 were subsequently quantified.

(G-H) Statistical graph of FIG7I and FIG7J. METTL14 was knocked out in PANC-1 and BxPC-3 cells first. Then cells were then cultured at 20 kPa for 10 days (to induce mechanical memory formation) or 3 days (without mechanical memory formation), followed by transfer to a 1 kPa culture condition for 5 days, and the expression level of CD166 and YAP1 were subsequently quantified.

(I-J) Statistical graph of FIG7K and FIG7L. METTL14 was knocked out in PANC-1 and BxPC-3 cells first. Then cells were then cultured at 20 kPa for 10 days (to induce mechanical memory formation) or 3 days (without mechanical memory formation), followed by transfer to a 1 kPa culture condition for 5 days,The expression level of SOX2, NANOG and OCT4 were subsequently quantified.

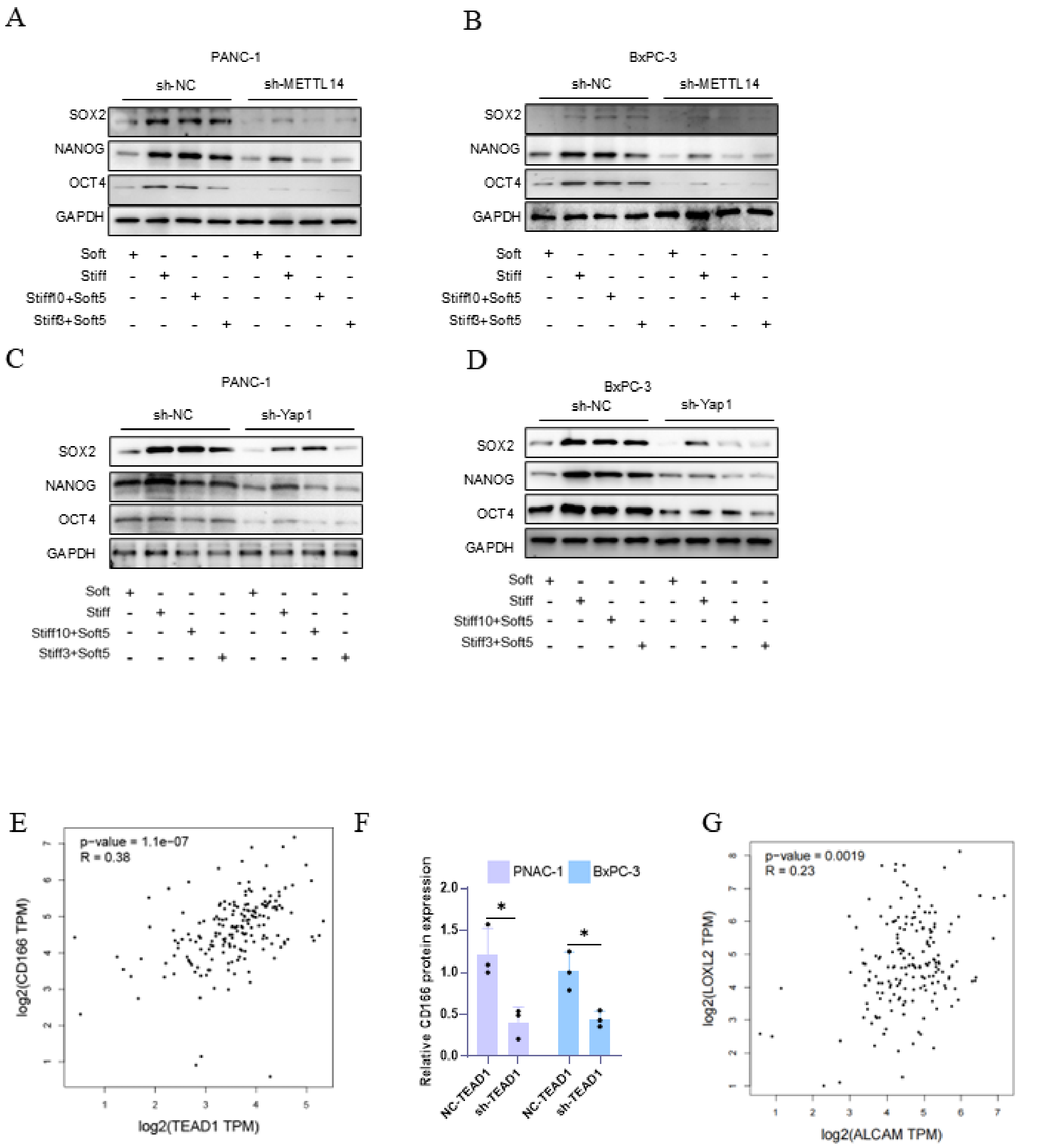

Fig. S5

(A-B) METTL14 was knocked out in PANC-1 and BxPC-3 cells first. Then cells were then cultured at 20 kPa for 10 days (to induce mechanical memory formation) or 3 days (without mechanical memory formation), followed by transfer to a 1 kPa culture condition for 5 days, and the expression level of CD166, SOX2, NANOG and OCT4 were detected by western blotting.

(B-D) METTL14 was knocked out in PANC-1 and BxPC-3 cells first. Then cells were

then cultured at 20 kPa for 10 days (to induce mechanical memory formation) or 3 days (without mechanical memory formation), followed by transfer to a 1 kPa culture condition for 5 days, and the expression level of CD166, SOX2, NANOG and OCT4 were detected by western blotting.

(E)TCGA analysis elucidated the correlation between CD166 and TEAD1 in pancreatic cancer.

(F) Statistical graph of FIG7N. Alterations in CD166 expression levels detected via western blot following TEAD1 knockdown, and the expression level of CD166 were subsequently quantified.

(G) TCGA analysis elucidated the correlation between CD166 and LOXL2 in pancreatic cancer.

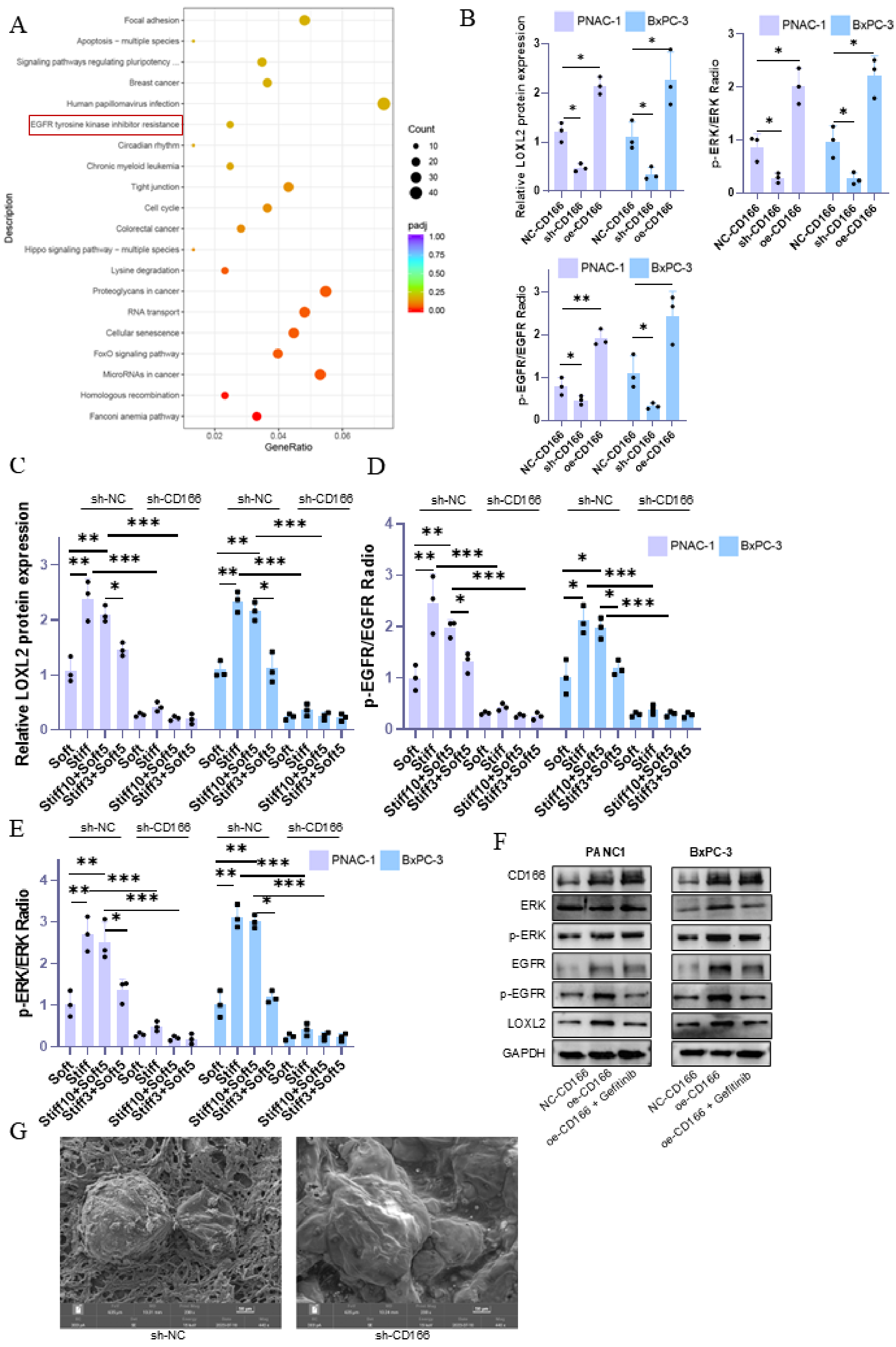

Fig. S6

(A)KEGG enrichment analysis results of BxPC-3 cells cultured under 1 kPa vs 20 kPa

(B) Statistical graph of FIG7P. Western blot analyses detailing alterations in the EGFR pathway and LOXL2 expression following CD166 knockdown or overexpression. The expression level of CD166, p-EGFR/EGFR radio and p-ERK/ERK radio were subsequently quantified.

(C-E) Statistical graph of FIG7Q and FIG7R. CD166 was knocked out in PANC-1 and BxPC-3 cells first. Then cells were then cultured at 20 kPa for 10 days (to induce mechanical memory formation) or 3 days (without mechanical memory formation), followed by transfer to a 1 kPa culture condition for 5 days, and the expression level of CD166, p-EGFR/EGFR radio and p-ERK/ERK radio were subsequently quantified.

(F) Alterations in the EGFR pathway and LOXL2 expression subsequent to CD166 overexpression and gemcitabine treatment.

(G) SEM results of matrix cross-linked cell surfaces in NC and sh-CD166 pancreatic cancer BxPC-3 cells.
